# Syngeneic animal models of tobacco-associated oral cancer reveal the activity of in situ anti-CTLA-4

**DOI:** 10.1038/s41467-019-13471-0

**Published:** 2019-12-05

**Authors:** Zhiyong Wang, Victoria H. Wu, Michael M. Allevato, Mara Gilardi, Yudou He, Juan Luis Callejas-Valera, Lynn Vitale-Cross, Daniel Martin, Panomwat Amornphimoltham, James Mcdermott, Bryan S. Yung, Yusuke Goto, Alfredo A. Molinolo, Andrew B. Sharabi, Ezra E. W. Cohen, Qianming Chen, J. Guy Lyons, Ludmil B. Alexandrov, J. Silvio Gutkind

**Affiliations:** 1 Moores Cancer Center, University of California San Diego, 3855 Health Sciences Drive, La Jolla, CA USA; 2 Department of Pharmacology, University of California San Diego, La Jolla, CA USA; 3 Department of Cellular and Molecular Medicine, University of California San Diego, La Jolla, CA USA; 4 Cancer Biology and Immunotherapies Group, Sanford Research, Sioux Falls, SD USA; 5 National Institute of Dental and Craniofacial Research, National Institutes of Health, Bethesda, MD USA; 6 International College of Dentistry, Walailak University, Nakhon Si Thammarat, Thailand; 7 Department of Radiation Medicine and Applied Sciences, University of California San Diego, La Jolla, CA USA; 8 Department of Medicine, Division of Hematology-Oncology, University of California, San Diego, La Jolla, CA USA; 9 State Key Laboratory of Oral Diseases, National Clinical Research Center for Oral Diseases, West China Hospital of Stomatology, Sichuan University, Chengdu, China; 10 Dermatology, Bosch Institute, University of Sydney, Camperdown, NSW 2050 Australia; 11 Cancer Services, Royal Prince Alfred Hospital, Camperdown, NSW 2050 Australia; 12 Centenary Institute, Camperdown, NSW 2050 Australia

**Keywords:** Cancer, Oral cancer, Immunology

## Abstract

Head and neck squamous cell carcinoma (HNSCC) is the sixth most common cancer worldwide. Tobacco use is the main risk factor for HNSCC, and tobacco-associated HNSCCs have poor prognosis and response to available treatments. Recently approved anti-PD-1 immune checkpoint inhibitors showed limited activity (≤20%) in HNSCC, highlighting the need to identify new therapeutic options. For this, mouse models that accurately mimic the complexity of the HNSCC mutational landscape and tumor immune environment are urgently needed. Here, we report a mouse HNSCC model system that recapitulates the human tobacco-related HNSCC mutanome, in which tumors grow when implanted in the tongue of immunocompetent mice. These HNSCC lesions have similar immune infiltration and response rates to anti-PD-1 (≤20%) immunotherapy as human HNSCCs. Remarkably, we find that >70% of HNSCC lesions respond to intratumoral anti-CTLA-4. This syngeneic HNSCC mouse model provides a platform to accelerate the development of immunotherapeutic options for HNSCC.

## Abstract (web summary)

Tobacco use is one of the major risk factors for Head and neck squamous cell carcinoma (HNSCC). Here, the authors report an immune-competent syngeneic mouse model that mimics human tobacco-related HNSCC, and develops tumors in the tongue, and report a high response rate to anti-CTLA-4 therapy.

## Introduction

Tobacco smoking claims the lives of more than 6 million people every year worldwide and is the leading cause of cancer deaths in the US^1,2^. Tobacco use has been associated with at least 17 types of cancer, primarily in the lung, as well as with carcinomas arising in the oral cavity, pharynx, and larynx, often referred to as squamous cell carcinomas of the head and neck (HNSCC)^1,2^. HNSCC is a significant public health issue, with more than 65,400 new cases resulting in 14,600 deaths in 2019 in the US. alone^3^. The main risk factors include tobacco use and human papillomavirus (HPV) infection, the latter of which is predicted to diminish in the future due to successful vaccination campaigns^4,5^. Depending on the stage of disease, HNSCC is typically treated with surgery, radiotherapy, chemotherapy, or a combination of these interventions. These standard therapies result in a 5-year survival of ~63%, but patients with more advanced stages have higher rates of mortality (5 year survival <50%) and require multimodality treatments, which can lead to occurrence of significant long-term side effects and lower quality of life^6,7^. Despite this more aggressive regimen, up to 30–60% of HNSCC patients develop tumor recurrence, and often succumb to the disease^8^. The recent elucidation of the genomic alterations underlying HNSCC progression and new immunotherapeutic strategies may provide an opportunity for the development of more effective treatment options for HNSCC.

Revolutionary breakthrough discoveries in cancer immunology have demonstrated that a patient’s own immune cells can be manipulated to target, attack, and destroy cancer cells^9–11^. A key emerging mechanism of tumor immune evasion involves T-cell exhaustion, whereby T-cell reactivity is impaired due to activation of T-cell checkpoints, including PD-1 by its ligand, programmed death ligand 1 (PD-L1) that is expressed by macrophages and some cancer cells, including HNSCC, restraining T-cell activation (reviewed in ref. ^12^). Indeed, immune checkpoint blockade (ICB) by new immunotherapeutic agents such as pembrolizumab and nivolumab (anti-PD-1) have recently demonstrated potent antitumor activity in a subset of HNSCC patients^13–16^. However, 1-year survival and response rates of anti-PD-1 in HNSCC were only 36 and 14%, respectively, which highlights the urgent need to identify novel therapeutic options to increase the effectiveness of ICB for >80% of patients that do not have an objective response to anti-PD-1/PD-L1 treatment^13–15^.

Animal models with a full functioning immune system that also properly resemble human HNSCC etiology and mutational landscape are desperately needed to accurately recapitulate the complexity of the tumor immune microenvironment (TIME), thereby accelerating the search for new immune therapeutic options. Here, we report a syngeneic murine HNSCC cell panel that recapitulates typical human tobacco-related HNSCC genomic alterations and mutational landscape, and we show that these cells form squamous carcinomas (HNSCC) when implanted orthotopically in the tongue of immune-competent C57Bl/6 mice. These HNSCC lesions have immune infiltration and response rates to anti-PD-1 therapies (≤20%) similar to those of human HNSCCs, thereby providing a platform for the evaluation of new immune oncology (IO) options for HNSCC treatment.

## Results

### 4MOSC models exhibit tobacco-related genomic landscapes

**Fig. 1:**
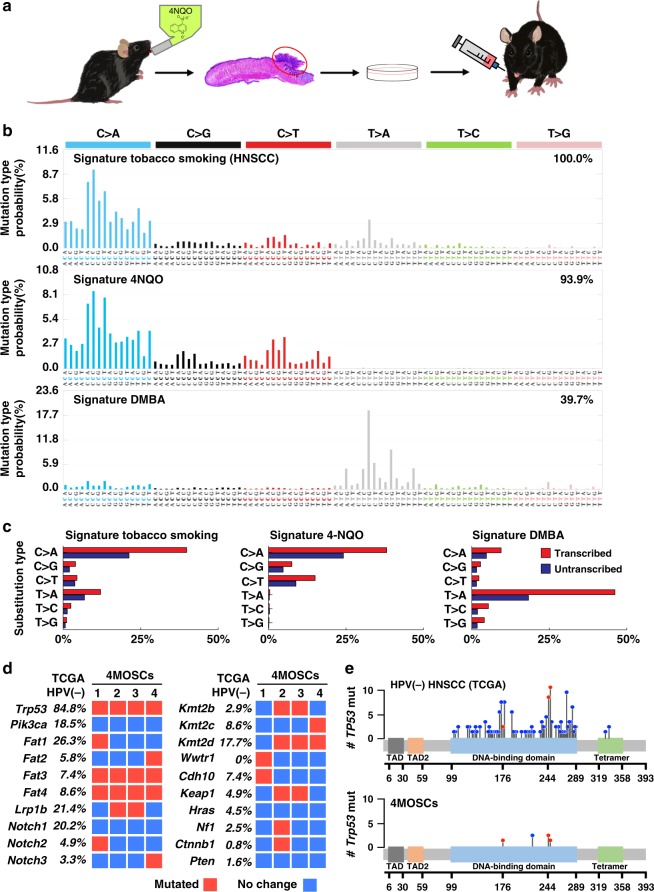
Development of a novel syngeneic mouse model for oral squamous cell carcinoma. **a** Experimental scheme of 4NQO syngeneic model. C57Bl/6 mice were given 4NQO (50 μg/mL) in the drinking water for 16 weeks and then regular water until week 22. Cells were isolated from the lesions, cultured, and then implanted into the tongue of wild-type C57Bl/6 mice. The Scheme was drawn by Yagi and Allevato. **b** Mutational signatures associated with tobacco smoking. The somatic mutational profiles of the four lesions from mice exposed to 4NQO were correlated to known mutational signatures in human cancer (Pearson correlation > 0.93)^19,24^. Top, Signature 4 extracted from cancers associated with tobacco smoking, this signature was found only in cancer types in which tobacco smoking increases risk and mainly in those derived from epithelia directly exposed to tobacco smoke^19^; middle, the pattern of a mutational signature of lesions from mice exposed to 4NQO, compilation of all four samples analyzed; bottom, the pattern of a mutational signature of lesions from mice exposed to DMBA. The similarity between signature tobacco smoking associated HNSCC and signature 4NQO is 93.9%; and the similarity between signature tobacco smoking associated HNSCC and signature DMBA is only 39.7%. **c** Percentage of somatic substitutions located in translated or untranslated in tobacco smoking associated HNSCC patients (left), 4NQO derived lesions (middle) and DMBA derived lesions (right). **d** Graphical matrix representation of the individual mutations in four syngeneic cell lines (4MOSCs) isolated from lesions from mice exposed to 4NQO. Listed are the alterations most frequently observed in human HNSCC and the corresponding percentage of mutations. Mutations (red), or no mutations (blue) are listed in rows and four different cell lines are in column. **e** Mutational plot of *TP53* mutations in 243 HPV-negative tumor samples from TCGA (top) and of four syngeneic cell lines (4MOSCs) (bottom). Frequency of mutation is depicted by height of lollipop, blue circles represent mutations unique to human or mouse, and red circles depict mutations in common between human and mouse HNSCCs.

Tobacco smoke contains a number of harmful carcinogens that drive tumorigenesis, the exposure to which strongly correlates with cancer incidence^17^.While tobacco-associated cancers are generally characterized by high mutation frequencies^18^, we have recently reported that they can be defined by very specific set of mutational signatures^19^. We have also described the optimization of a carcinogen-induced oral cancer mouse model in which the compound 4-nitroquinoline-1 oxide (4NQO), a DNA adduct-forming agent that causes DNA damage and can act as a tobacco-mimetic promoting *Tp53* mutations and oral cancer initiation and progression^20^. This model has been used extensively to study HNSCC progression and preventive and treatment therapeutic options^21–23^. However, its direct relevance to the mutagenic process in human HNSCC has not been previously established. To begin developing syngeneic HNSCC animal models, we first isolated four representatives murine HNSCC cell lines from primary 4NQO-induced tumors in the tongue of C57Bl/6 mice (designated 4MOSC1-4, short for 4NQO-induced murine oral squamous cells) (Fig. 1a). The use of SigProfiler^24,25^ to analyze exome DNAseq of these HNSCC cells revealed a remarkable 93.9% similarity with human cancer signature 4, which is strictly associated with tobacco smoking, including in HNSCC, esophageal cancer, and lung cancer^19^ (Pearson correlation >0.93) (Fig. 1b and individual 4MOSC cells in Supplementary Fig. 1). This similarity between 4NQO-induced mutational patterns and tobacco extended to the presence of a transcriptional strand bias (Fig. 1c), which reflects the rate of substitution type on each nucleotide. In contrast, the mutational signature of SCC caused by DMBA, a carcinogen found in tobacco smoke that is the most widely used agent for experimental carcinogenesis studies^26^, showed only 39.7% similarity with human cancer signature 4. This suggests that 4NQO-induced SCC lesions better mimic human tobacco-related human HNSCC. Indeed, these cells also exhibit typical HNSCC histology and mutations impacting *Trp53*, *Fat1-4*, *Keap1*, *Notch1-3*, *Kmt2b-d*, and others, which represent some of the most frequently altered gene pathways in HPV− human HNSCC (Fig. 1d, e, and Supplementary Data 1–4). Of note, similar to HPV(−) HNSCC samples from The Cancer Gene Atlas (TCGA), all four 4MOSC cells exhibit typical inactivating *Trp53* mutations in its core DNA binding domain, including hot spot residues (G245, and R248) that result in loss of tumor-suppression and gain of tumorigenesis and invasiveness^27^.

### 4MOSC lesions mimic the human HNSCC immune microenvironment

**Fig. 2:**
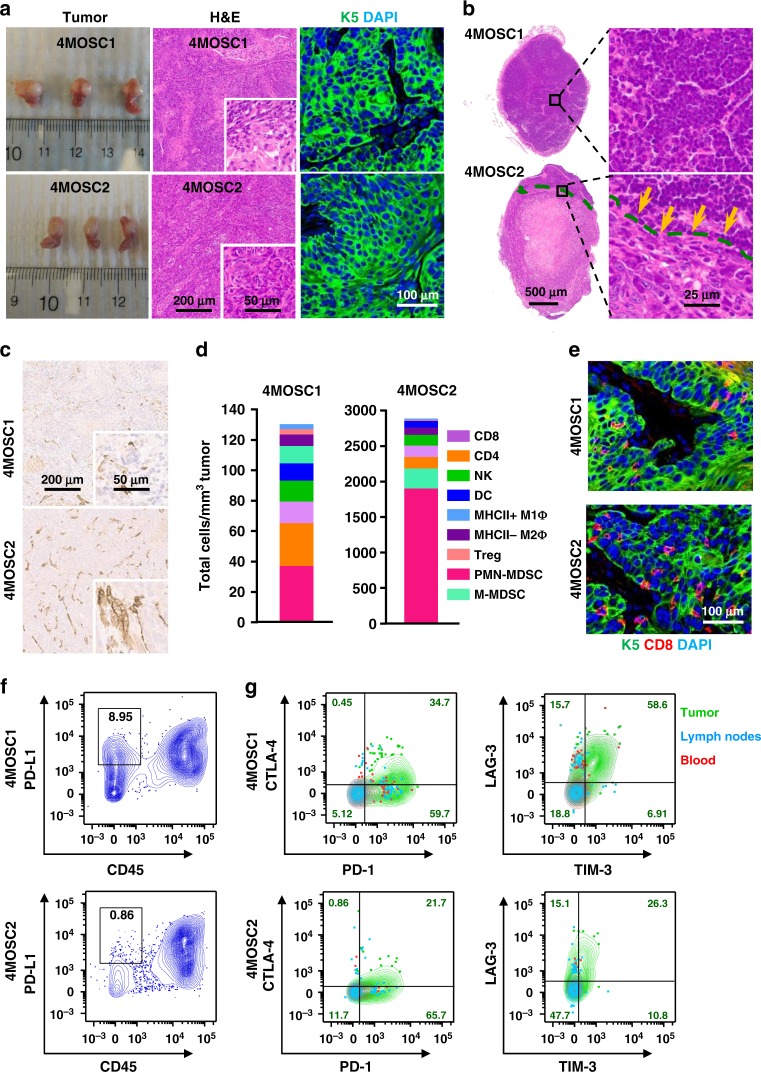
Characterization of 4NQO-induced murine oral squamous cell model. **a** Left panel, C57Bl/6 mice were implanted with 1 × 10^6^ of either 4MOSC1 or 4MOSC2 cells into the tongue. Tongue lesions when the tumor volume reached ~100 mm^3^. Middle panel, representative H&E staining of histological tissue sections from mouse tongues with 4MOSC1 or 4MOSC2 tumors. Right, representative pictures of tumors stained to show expression of cytokeratin 5 (CK5, green) and DAPI (blue) (*n* = 3 mice per group). **b** Top panel, representative H&E stain of a nonmetastatic cervical lymph node from mice with 4MOSC1 tumors. Bottom panel, representative H&E stain of a metastatic cervical lymph node from mice with 4MOSC2 tumors. Metastatic growth of 4MOSC2 cells into the lymph node is depicted with a dotted line in the bottom area (*n* = 5 mice per group). **c** Representative tumor tissue sections stained for LYVE-1 by immunohistochemistry in 4MOSC1 or 4MOSC2 tumors (*n* = 3 mice per group). **d** Absolute number of immune cells infiltrating 4MOSC1 or 4MOSC2 tumors. Shown is the average number of live cells infiltrating per mm^3^ of tumor (*n* = 3 mice per group). **e** Immunofluorescent staining of CK5 and CD8 to show squamous cell character of the lesion and CD8 infiltration in mice with 4MOSC1 or 4MOSC2 tumors, respectively (*n* = 3 mice per group) (CK5, green; CD8, red; DAPI, blue). 4MOSC1 or 4MOSC2 tumors were isolated from mice and mechanically and enzymatically digested. Single-cell suspension was then stained with CD45, Nk1.1, CD3, CD8, CD44, PD-L1, PD-1, CTLA-4, LAG-3, and TIM-3 fluorescent labeled antibodies and analyzed by flow cytometry. Shown are representative flow cytometry plots of **f** the frequency of tumor cells (CD45 negative) expressing PD-L1 and **g** the frequency of CD8^+^/CD44^+^ cells expressing inhibitory receptors PD-1, CTLA-4, LAG-3, and TIM-3 in individual tumors (*n* = 4 mice per group). Contour plots of lymphocytes from tumor (green), and corresponding cervical lymph nodes (blue), and blood (red) are overlaid and the frequencies of tumor CD8^+^/CD44^+^ T cells expressing each inhibitory receptor are shown (*n* = 4 mice per group).

Transplantation of the 4MOSC cells orthotopically into the tongue of immunocompetent C57Bl/6 mice led to the formation of well-differentiated HNSCC tumors in two of the cell lines, 4MOSC1 and 4MOSC2, which exhibit typical HNSCC histology, as indicated by hematoxylin and eosin (H&E) stained sections and fluorescence cytokeratin 5 staining (Fig. 2a and Supplementary Fig. 2a). 4MOSC3 and 4MOSC4 cells also formed tumors, but they regressed spontaneously after 2 weeks, likely due to their rejection by the host immune system. Thus, we focused our studies on 4MOSC1 and 4MOSC2, with emphasis on investigating whether they have distinct biological properties reflecting human HNSCC. In this regard, since HNSCC has a high propensity to metastasize to locoregional lymph nodes (reviewed in ref. ^28^), leading to poor prognosis, we next addressed the metastatic potential of our model. Histological evaluation in H&E stained sections revealed growth of cancer cells in the lymph nodes of mice bearing 4MOSC2 but not 4MOSC1 tumors (Fig. 2b). Interestingly, locoregional lymph node invasion was observed as early as 2 days postimplantation; and a higher rate of lymph node metastasis was observed 8 days after 4MOSC2 tumors were established (Supplementary Fig. 2b). 4MOSC2 tumors also exhibited much higher density of lymphatic vessels staining positive for LYVE-1 than in 4MOSC1 (Fig. 2c), which is aligned with the strong correlation between intratumoral (IT) lymphangiogenesis and metastasis in human HNSCC (reviewed in ref. ^29^).

By flow cytometry analysis, we found that the immune cells infiltrating the TIME comprises of cytotoxic T cells (CD8), helper T cells (CD4), regulatory T cells (Treg), natural killer cells (NK), macrophages (M1Φ and M2Φ), as well as polymorphonuclear myeloid-derived suppressor cells, and monocytic myeloid-derived suppressor cells^30^ (Fig. 2d). Notably, although the ratios of immune infiltration were similar, 4MOSC2 tumors had a considerably higher level of infiltration than 4MOSC1 (Fig. 2d). Though cytotoxic CD8 T cells infiltrate both tumors at similar proportions relative to other immune cells, immunofluorescence staining showed more abundant distribution within 4MOSC2 tumor cells (Fig. 2e). The more immune inflamed state of 4MOSC2 tumors is likely due to their clearly distinct chemokine and cytokine profile. Indeed, 4MOSC2 tumors express higher levels of multiple chemokines (including CXCL1 and CXCL5) and growth factors (such as G-CSF, GM-CSF) than in 4MOSC1 tumors, which may contribute to the recruitment and survival of MDSCs and inflammatory cells, as well as VEGF that may explain the higher density of lymphatic vessels **(**Supplementary Fig. 3**)**.

To determine whether this immune infiltration is associated with antigen-driven immunogenicity, we next investigated whether the 4MOSC tumors could generate memory immune responses. Initial exposure of mice to tumor cell antigens was achieved by first irradiating tumor cells and then injecting into the tongue of C57Bl/6 mice with or without polyinosinic-polycytidylic acid (poly IC) as an immune adjuvant. Irradiated 4MOSC1 and 4MOSC2 cells did not form tumors, and mice vaccinated with irradiated tumor cells alone or irradiated tumor cells with poly IC failed to form tumors when they were subsequently rechallenged with nonirradiated cancer cells, while naïve mice and mice with poly IC alone still formed tumors. This suggests that the mice were able to develop an immunological memory to 4MOSC antigens even in the absence of an immune adjuvant. This may be due to the fact that irradiation can induce inflammatory cell death^31^, which contributes to immunogenicity. Taken together, our results suggest that these syngeneic HNSCC cell lines are highly immunogenic (Supplementary Fig. 4).

These findings indicate that these mice are capable of generating adaptive immune responses against 4MOSC tumor antigens. However, 4MOSC tumors still grow and lead mice to succumb to disease, implying that these tumors can evade immunity by inducing an immune suppressive microenvironment. The expression of PD-L1, the ligand for the T-cell inhibitory receptor PD-1, is often high in HNSCC patients (46–100% of tumors)^32^ and has been shown to suppress cytotoxic T cells that destroy tumors and also serves as a biomarker (reviewed in ref. ^33^) predicting a better response to anti-PD-1 therapy. We found that PD-L1 is constitutively expressed on both tumor (CD45^−^) and immune cells (CD45^+^), but the frequency of 4MOSC1 tumor cells that expressed PD-L1 was much higher than the frequency of 4MOSC2 cells expressing PD-L1 (Fig. 2f). Though most tumor-infiltrating immune cells expressed PD-L1, MDSCs, tumor-associated macrophages and MHCII^+^ antigen-presenting cells infiltrating the tumor comprise of the majority of PD-L1^hi^CD45^+^ immune cells (Supplementary Fig. 5). Activated (CD44^+^) CD8 T cells infiltrating both 4MOSC1 and 4MOSC2 tumors exhibited characteristic immune checkpoint molecules, PD-1, cytotoxic T lymphocyte associated protein 4 (CTLA-4), T-cell immunoglobulin mucin 3 (TIM-3), and lymphocyte activation gene 3 (LAG-3) (Fig. 2g). Interestingly, there were higher immune checkpoint molecules in tumor-infiltrating lymphocytes (TILs, green) than in lymph nodes (blue) or blood (red) in both tumors (Fig. 2g). Similar expression patterns were seen for tumor-infiltrating CD4 T cells, but there was a higher frequency of Tregs expressing CTLA-4 compared with non-Treg CD4 T cells (Supplementary Fig. 6).

### Limited response of syngeneic HNSCC tumors to PD-1 blockade

**Fig. 3:**
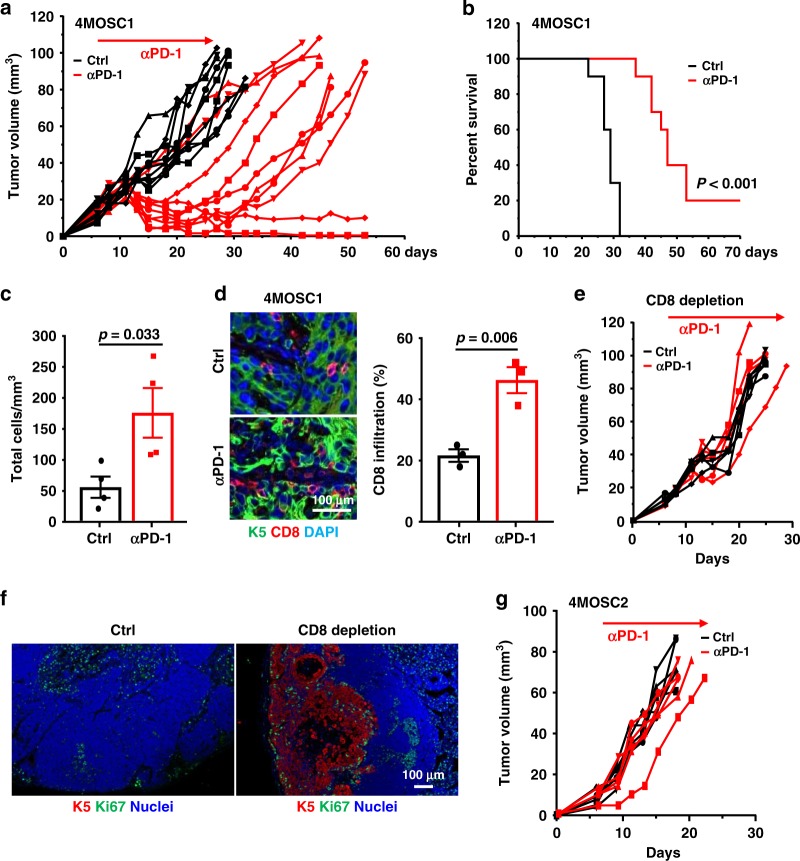
Variable responses to anti-PD-1 in mice with 4MOSC1 tumors. **a** C57Bl/6 mice were implanted with 1 × 10^6^ of 4MOSC1 cells into the tongue. After tumors reached ~30 mm^3^, mice were treated IP with 10 mg/kg of isotype control or anti-PD-1 (*n* = 10 per group). Individual growth curves of 4MOSC1 tumor-bearing mice are shown. **b** A Kaplan–Meier curve showing the survival of mice from **a**. The death of animals occurred either naturally, when tumor compromised the animal welfare, or when tumor volume reached 100 mm^3^ (*n* = 10 mice per group; Log-Rank/Mantel–Cox test.). **c** Absolute number of live CD45^+^CD3^+^CD8^+^ T cells infiltrating 4MOSC1 tumors with or without anti-PD-1 treatment. Shown is the average of the number of live CD8 T cells infiltrating per mm^3^ of tumor (*n* = 4 mice per group; two-sided Student’s *t* test; data are represented as mean ± SEM). **d** Immunofluorescent staining of CD8 highlights an increase in CD8 T-cell recruitment with anti-PD-1 treatment. Shown is the average CD8 positivity was by three regions of interest (ROI) per mouse (*n* = 3 mice per group; two-sided Student’s *t* test; data are represented as mean ± SEM). **e** Dependency of anti-PD-1 on CD8 T cells. C57Bl/6 mice were treated with CD8 T cell depleting antibody daily for 3 days before tumor implantation and then once a week after. Mice were then implanted with 1 × 10^6^ of 4MOSC1 cells into the tongue. After tumors reached ~30 mm^3^, mice were treated IP with 10 mg/kg isotype control or 10 mg/kg anti-PD-1 (*n* = 5 per group). Individual growth curves of 4MOSC1 tumor-bearing mice are shown. **f** Immunofluorescence staining of CK5 and Ki-67 in cervical lymph nodes of control or CD8-depleted 4MOSC1-bearing mice. Metastatic lesions in the lymph nodes showed abundant Ki-67^+^ proliferating tumor cells (*n* = 5 mice per group). **g** C57Bl/6 mice were implanted with 1 × 10^6^ of 4MOSC2 cells into the tongue. After tumors reached ~30 mm^3^, mice were treated IP with 10 mg/kg isotype control or 10 mg/kg anti-PD-1 (*n* = 5 per group). Individual growth curves of 4MOSC2 tumor-bearing mice are shown.

To interrogate whether blocking the interaction of PD-1 and PD-L1 could cause tumor regression, we first studied 4MOSC1 tumors, the syngeneic HNSCC cell line that has higher PD-L1 expression. Most mice showed an initial decreased tumor volume after anti-PD-1 treatment (Fig. 3a), with a consequent increased overall survival (Fig. 3b) (*p* < 0.001). Interestingly, however, 80% of mice that initially responded to anti-PD-1 (partial response) showed tumor relapse and eventually succumbed to disease burden, while 10–20% of the mice showed complete responses (CR) (Fig. 3a, b, and Supplementary Fig. 7a). Due to the limited tumor size and number of infiltrating cells, we could not perform a comprehensive analysis of immune cell infiltration in individual tumors at the beginning of the treatment. However, 4MOSC1 tumors from mice treated with anti-PD-1 showed clearly significant higher CD8 infiltration (*p* < 0.01) compared with tumors from untreated mice by FACS analysis of TILs and by immune fluorescence analysis of treated tissues (Fig. 3c, d, respectively). All responses to anti-PD-1 were abolished if CD8 T cells were eliminated from mice (Fig. 3e and Supplementary Fig. 7b). Together, these data indicate a CD8-dependent anti-PD-1 response in mice with 4MOSC1 tumors, but with limited durable disease control or tumor regression, which is similar to the clinical response to anti-PD-1 therapies in HNSCC patients^14,16^. To our surprise, although metastasis was not observed in 4MOSC1 control mice (see above), the cervical lymph nodes of CD8-depleted mice with 4MOSC1 tumors showed tumor invasion, as indicated by cytokeratin 5 staining (Fig. 3f) and visualization with H&E staining (Supplementary Fig. 7c) suggesting that immune surveillance may prevent the metastatic spread of this tumor. In contrast, mice bearing 4MOSC2 tumors failed to respond to anti-PD-1 treatment (Fig. 3g and Supplementary Fig. 7d), suggesting 4MOSC2 serves as a less differentiated, high immune cell-infiltrated, metastatic, and PD-L1-low, and anti-PD-1 resistant model (Fig. 3g, and Supplementary Fig. 7d).

The PD-1 blocking antibodies used are mouse specific, hence treatment failure is not expected to be due to neutralizing antibodies against human IgG. We next isolated 4MOSC1 cells from mice showing limited response to anti-PD-1 as an approach to achieve a more clear perspective of immune suppressive mechanisms that may account for the recurrence and progression of 4MOSC1 tumors in mice treated with anti-PD-1. We then engrafted sensitive (parental) and anti-PD-1 (αPD-1) resistant 4MOSC1 cells and treated mice with or without anti-PD-1. Among all immune cells examined, αPD-1 resistant tumors recruited significantly more Ly6-G^hi^ PMN-MDSCs than the parental cell line, and treatment with anti-PD-1 in these αPD-1 resistant tumors significantly increased immunosuppressive PMN-MDSCs, suggesting that MDSCs may be deterring cytotoxic immune responses. Moreover, upon anti-PD-1 treatment, there were significant increases in LAG-3 and TIM-3, and greater increase in CTLA-4 in CD8 T cells isolated from the αPD-1 resistant tumors when compared with their parental tumors, while increases in LAG-3 in CD4 T cells were comparable between parental and αPD-1 resistant tumors (Supplementary Fig. 8). Altogether, these data suggest that recurrence and acquired resistance to anti-PD-1 in 4MOSC1 tumors may be conferred by increased MDSC recruitment and higher expression of additional CD8 T-cell inhibitory receptors.

### Immune modulation by intratumoral (in situ) delivery of ICB

**Fig. 4:**
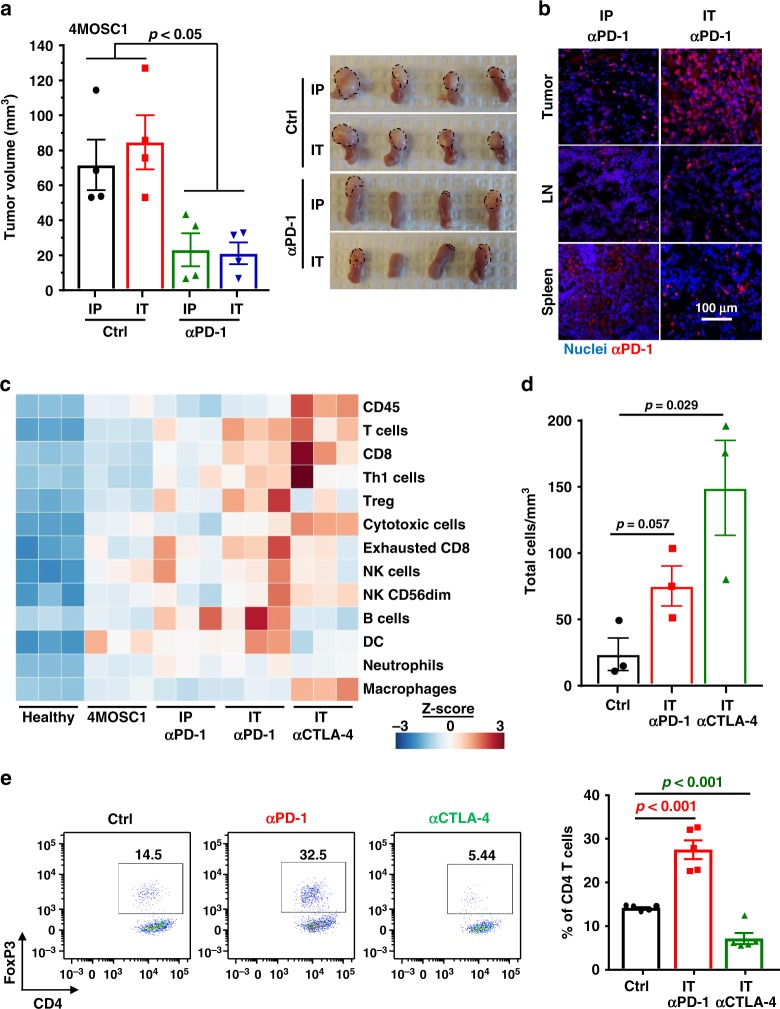
Efficacy of intratumoral delivery of immune oncology agents. **a** Left panel, C57Bl/6 mice were implanted with 1 × 10^6^ of 4MOSC1 cells into the tongue. After tumors reached ~30 mm^3^, mice were either treated IP or by intratumoral (IT) delivery of PBS, IP with 10 mg/kg or IT with 5 mg/kg anti-PD-1. Shown is the average volume of each tumor (*n* = 4 mice per group; two-sided Student’s *t* test; data are represented as mean ± SEM). Right panel, representative pictures of tongues from mice in **a** with tumors depicted with a dotted line. **b** Distribution of anti-PD-1 antibody in mice with 4MOSC1 tumors using IP or IT delivery of the treatment. Staining for anti-hamster IgG showed the localization of anti-PD-1 antibody in the tongue, lymph nodes, and spleen of treated mice (*n* = 4 mice per group). **c** RNA from each tumor was isolated and comprehensive immune profiling was analyzed using the NanoString nCounter PanCancer Mouse Immune Profiling gene expression platform. The advanced analysis module of the nSolver software was used to analyze genes associated with listed immune cells and given a score. Shown is the *Z*-score of each cell profile score (*n* = 3 mice per group). **d** Absolute number of live CD45^+^CD3^+^CD8^+^ T cells infiltrating 4MOSC1 tumors with or without anti-PD-1 or anti-CTLA-4 treatment. Shown is the average of the number of live CD8 T cells infiltrating per mm^3^ of tumor (*n* = 3 mice per group; two-sided Student’s *t* test; data are represented as mean ± SEM). **e** Frequency of live CD45^+^CD3^+^CD4^+^ FoxP3^+^ Tregs infiltrating 4MOSC1 tumors with or without anti-PD-1 or anti-CTLA-4 treatment. Left panel, a representative flow cytometry plot from one mouse showing the frequency of Tregs (CD4^+^FoxP3^+^) out of CD4^+^ cells is shown. Right panel, the frequency of Tregs out of CD4^+^ cells was quantified following treatment with anti-PD-1 or anti-CTLA-4 (*n* = 5 mice per group; two-sided Student’s *t* test; data are represented as mean ± SEM).

A defining feature of most HNSCCs is the superficial and mucosal localization of the disease. Unlike many other cancer types, most HNSCC patients have tumors that can be readily visualized and accessed by surgeons, providing an opportunity to use IT drug delivery. To investigate whether IT injection of anti-PD-1 has improves activity in our model, we compared the effectiveness of using a lower dose anti-PD-1 treatment with standard systemic delivery. We found that mice treated with just half the dose of anti-PD-1 locally showed similar antitumor responses compared with mice with full dose systemic treatment (Fig. 4a). Furthermore, immunofluorescence analysis revealed that IT delivery led to significantly higher PD-1 antibody distribution in tumors and cervical lymph nodes and lower distribution in the spleen as a peripheral organ when compared with systemic delivery (Fig. 4b).

To investigate if there are local, differential immune signature alterations associated with IT drug delivery, we performed a comprehensive immune profiling using the nCounter PanCancer Mouse Immune Profiling gene expression platform (NanoString Technologies). Using 770 immune-related genes, we profiled immune cells infiltrating the tumor following treatment with systemic or IT anti-PD-1, and compared it with another FDA-approved immunotherapy, CTLA-4 blockade. Relative to the tongues from healthy mice, mice bearing 4MOSC1 tumors have elevated expression of a majority of immune cell-associated genes. While systemic anti-PD-1 treatment increased the T-cell signature, IT delivery enhanced it further while also increasing gene expression of NK and genes related to cytotoxic immune cells (Fig. 4c). Interestingly, albeit used initially as a control, this analysis revealed that IT treatment with anti-CTLA-4 led to even more robust T cell, cytotoxic cell, and macrophage responses, and an increased CD8 T-cell tumor infiltration (Fig. 4c, d). In addition, while anti-PD-1 increased Treg associated gene signatures, anti-CTLA-4 appears to diminish it, which was confirmed by flow cytometry analysis of treated tumors (Fig. 4c, e). In order to further explore the role of Tregs in 4MOSC1 tumors, we utilized the *FoxP3*^DTR^ transgenic mice that have been widely used to study the immunosuppressive role of Tregs in cancer^34^. Treg depletion with diphtheria toxin (DT) led to significant reduction of tumor growth, and combination of Treg depletion with anti-PD-1 led to complete responses in most mice (Supplementary Fig. 9). This strongly suggests that Tregs may prevent the full therapeutic activity of anti-PD-1, and that the reduction of the immunosuppressive activity of Tregs may represent a mechanism explaining the higher therapeutic responses to anti-CTLA-4 treatment (Supplementary Fig. 9).

### The majority of 4MOSC1 lesions respond to anti-CTLA-4

**Fig. 5:**
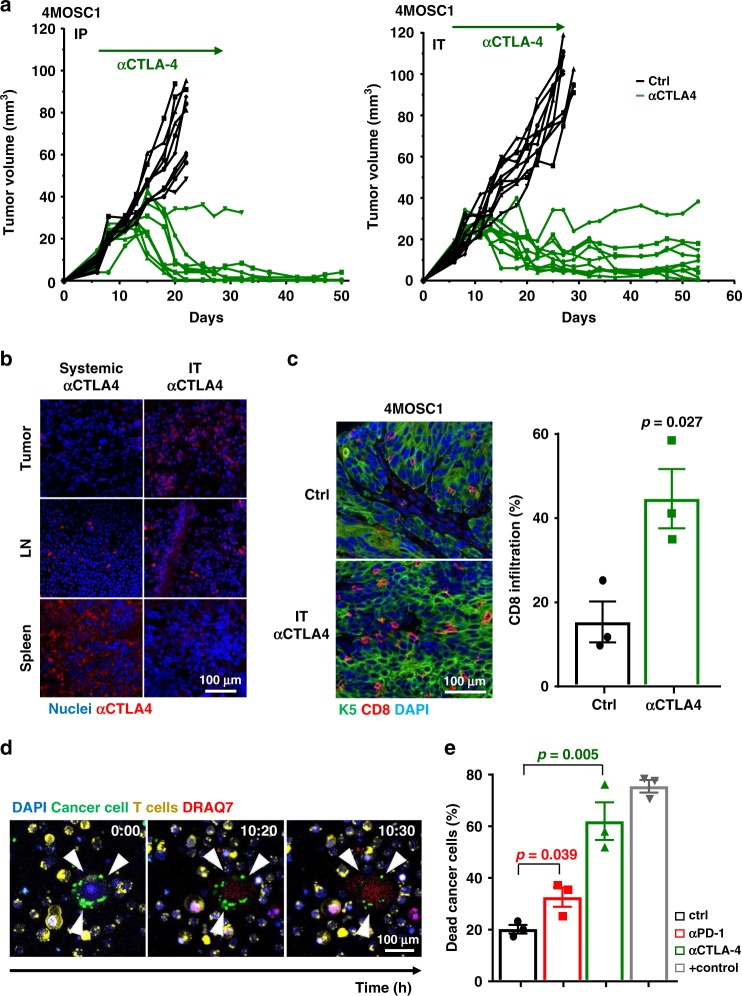
Mice with 4MOSC1 tumors show nearly complete response to anti-CTLA-4. **a** C57Bl/6 mice were implanted with 1 × 10^6^ of 4MOSC1 cells into the tongue. After the tumors reached ~30 mm^3^, mice were treated 10 mg/kg of isotype control or anti-CTLA-4 for IP administration (left), and 5 mg/kg of isotype control or anti-CTLA-4 for IT administration (right). Individual growth curves of 4MOSC1 tumor-bearing mice plotting primary tumor growth are shown (*n* = 10 mice per group). **b** Shown is the immunofluorescent staining of the distribution of anti-CTLA-4 antibody for mice with 4MOSC1 tumors using IP or IT delivery of the treatment. Staining for anti-hamster IgG (red) showed the localization of anti-CTLA-4 antibody in the tongue, lymph nodes, and spleen of treated mice. DAPI staining for nuclei is shown in blue (*n* = 4 mice per group). **c** Immunofluorescent staining of CD8 highlights an increase in CD8 T-cell recruitment with anti-CTLA-4 treatment. Quantification of CD8 T cells with or without anti-CTLA-4 treatment was done by immunofluorescent staining of tumor (CK5) in the tongue. Shown is the average CD8 positivity by three regions of interest (ROI) per mouse, quantified by Qupath software for each condition (*n* = 3 mice per group two-sided Student’s *t* test; data are represented as mean ± SEM). Antigen specific T-cell cytotoxic assay. C57Bl/6 mice were implanted with 1 × 10^6^ of 4MOSC1 cells into the tongue, and when they reached ~30 mm^3^, mice were treated IT with isotype control, anti-PD-1, or anti-CTLA-4 every other day for three treatments total. CD8 T cells from each group were isolated and cocultured with preplated 4MOSC1 cells. DMSO (10%) was used to treat 4MOSC1 as a positive control, and DRAQ7 was added in the culture medium to mark dead cells (red). **d** Real time live-imaging experiments were performed using the 880 confocal fast scan (Zeiss), and representative images of tumor cell killing (CD8 T cells from anti-CTLA-4 group) are shown at the indicated times. **e** Quantification of dead cancer cells at the end of experiment. (*n* = 3 mice per group; two-sided Student’s *t* test; data are represented as mean ± SEM).

Given that immune stimulatory effects were enhanced following anti-CTLA-4 treatment compared with anti-PD-1 treatment, we sought to determine whether mice with 4MOSC1 tumors can also respond to CTLA-4 blockade. CTLA-4 blockade systemically and IT elicited a robust antitumor effect, with 90% of the mice exhibiting a CR (Fig. 5a and Supplementary Fig. 10a) and efficiently resisted engraftment when rechallenged with fresh 4MOSC1 cells. Similar to anti-PD-1 treatment, IT delivery resulted in significantly higher anti-CTLA-4 antibody distribution in tumors and cervical lymph nodes and lower distribution in the spleen (Fig. 5b). Antitumor immunity of anti-CTLA-4 is also CD8 dependent, as CTLA-4 inhibition resulted in significantly increased infiltration of and IFNγ production by CD8^+^ T cells (Fig. 5c, Supplementary Fig. 11), and its antitumor activity was abolished by the depletion of CD8 T cells (Supplementary Fig. 10b). Adding to this, when tumor-infiltrating CD8 T cells were isolated and cultured with tumor cells in vitro, they were able to kill the tumor cells, and CD8 T cells from anti-PD-1- and anti-CTLA-4-treated mice were able induce significantly more cancer cell death (Fig. 5d, e). Of interest, 4MOSC2 tumors also failed to respond to anti-CTLA-4 treatment (Supplementary Fig. 10c), providing a model that is resistant to both forms of immunotherapy for future exploration of immunotherapy resistance and the use of strategic combinatorial modalities.

## Discussion

HNSCC is an immunosuppressive disease, in which the tumor deploys multiple mechanisms to evade immune surveillance and antitumor immune responses through the accumulation of immunosuppressive cytokines, impairment of cytotoxic activity and antigen-presenting function, and induction of T-cell exhaustion (reviewed in ref. ^12^). Based on this knowledge, numerous immunotherapeutic strategies were developed, including ICB, cancer vaccines, therapeutic cytokines, adoptive T-cell transfer, and adjuvants that may trigger innate immune responses such as TLR and STING agonists (reviewed in ref. ^12^). Clearly, suitable experimental systems that can model clinical responses are urgently needed to study and improve the effectiveness of IO approaches in HNSCC. Here, we developed a panel of C57Bl/6-derived syngeneic cells that resemble human HNSCCs closely with unique features: (i) The HNSCC cells have nearly identical tobacco-associated mutational signatures and genomic aberrations; (ii) they can be orthotopically transplanted into the tongue of immunocompetent C57Bl/6 mice; (iii) the tumors are histologically HNSCCs with abundant lymphangiogenesis and potential for lymph node metastasis; and (iv) the tumors exhibit abundant immune infiltration and are immunogenic, the latter as judged by their ability to induce immunological memory when used to vaccinate mice. These animal models may provide an opportunity to investigate the mechanisms driving intrinsic and acquired resistance to IO agents, as well as to identify novel therapeutic options increasing the response of currently available immunotherapies in HNSCC patients.

Tobacco use is one of the major risk factors for initiation and progression of HNSCC, and serves as an important prognostic factor for survival and mortality after cancer diagnosis (reviewed in ref. ^35^). In a recent study, we analyzed somatic mutations and DNA methylation in 5243 samples comprising of cancers for which tobacco smoking confers an elevated risk, which helped define the human tobacco-associated cancer signature^19^. Remarkably, although tobacco smoke is made up of thousands of chemicals, including more than 60 carcinogens^17^, we found that 4NQO exposure was sufficient to mimic the tobacco carcinogenic signature. This is supported by compelling evidence demonstrating that 4NQO-induced SCC lesions exhibit near identical association (~94%) with the tobacco mutational landscapes, recapitulating human HNSCC. This is in contrast with the mutational signature caused by DMBA (<40% similarity), which although representing a widely used tobacco carcinogen^26^, may not be as effective as 4NQO in reflecting the human tobacco-associated genetic signatures^19^.

In this regard, currently available syngeneic HNSCC models include SCCVII cells, HPV+ SCC cells designated MEER^36^ and a panel of mouse HNSCC cell lines from DMBA-treated mice (MOC1 and MOC2)^37^. Though widely used, SCCVII cells are in fact derived from a spontaneously formed skin SCC lesion in C3H mice^38^. MEER and MOC1/MOC2 models develop tumors in immune-competent C57Bl/6 mice when implanted in the flanks, which may not reflect the HNSCC TIME, albeit MOC1/MOC2 can also grow tumors orthotopically even if their immune status has not been characterized in this anatomical location^39^. These tumors are driven by *Ras* oncogenes (*Kras* in MEER and MOC2, and *Hras* in MOC1)^36,37,40^, which are very potent oncogenic drivers, but infrequently (<6%) mutated in human HNSCC^41^, albeit higher frequencies have been reported in some demographic groups^42^. Thus, although quite useful for cancer immunology studies, these cellular systems may not mimic fully the tobacco-induced carcinogenic process driving most human HNSCCs. Despite the fact that these cell lines have dramatically different genomic alteration profiles, some of these cells respond to ICB similarly to 4MOSC1 cells, suggesting that the determinants of immune responses may be independent of the underlying driving mutations that lead to HNSCC formation. Nonetheless, neither *Hras*, *Kras*, nor *Nras* genes are mutated in the 4MOSC panel, suggesting that these cells may harbor pathway specific alterations likely more relevant to human malignancy. Taken together, the unique features of our syngeneic HNSCC animal model provide a resource to investigate novel IO preclinical approaches for HNSCC treatment.

Seminal studies have shed light on T-cell exhaustion in human cancers, where CD8 T cells lose proliferative capacity, the ability to produce tumor necrosis factor (TNFα), interleukin-2, and interferon-γ (IFNγ), and upregulation of inhibitory checkpoint receptors, such as PD-1 and CTLA-4^9–11^. Recently, the successes of ICB to reverse T-cell exhaustion in multiple cancers illustrates the potential of therapeutic strategies targeting these negative regulatory pathways^43^. In the clinic, PD-1 blockade offers 10–20% clinical improvement in HNSCC^13–15^, which was modeled similarly in our study where anti-PD-1 led to regression of 4MOSC1 tumors in only ≤20% of mice. The increase in CD8 T cells seemed to provide only temporary cytotoxic activity in mice treated with anti-PD-1, as we observed the reoccurrence of tumors in the majority of treated mice. Surprisingly, we saw enhanced antitumor responses with anti-CTLA-4 treatment, where most 4MOSC1 tumor-bearing mice showed complete responses and no tumor reoccurrence. The resulting increase in CD8 T cells following anti-CTLA-4 treatment confirmed that targeting checkpoints may revitalize immunological effect of exhausted T cells, at least at the cellular level. One explanation for these strikingly different responses could be that PD-1 blockade may induce compensatory upregulation of FoxP3^+^ Treg cells^44,45^, as it occurred in our anti-PD-1-treated mice but not in anti-CTLA-4-treated mice. In fact, CTLA-4 inhibition led to significantly lower levels of FoxP3^+^ Treg cells in the tumors. In this regard, while both blocking antibodies can lead to cytotoxic CD8 T-cell responses, anti-CTLA-4 may provide additional antitumor immunity by depleting Tregs that mediate an immune-suppressive environment (reviewed in ref. ^46^). Moreover, PD-1 blockade predominantly activates T cells within the tumor, whereas anti-CTLA-4 may activate T cells primarily in the lymph nodes^46^, in which high levels of anti-CTLA-4 can be achieved by IT delivery. These and yet to be identified mechanisms may underlie the increased response to anti-CTLA-4 in some anti-PD-1 refractory HNSCC lesions, whose elucidation may provide biomarkers for the selection of patients that may benefit from anti-CTLA-4 treatment after failing to anti-PD-1 therapy.

In this regard, the recent CONDOR trial demonstrated no benefit to adding tremelimumab, a humanized monoclonal antibody against CTLA-4, to durvalumab, which blocks PD-L1, in patients with relapsed HNSCC^47^. However these are unique biological agents, as tremelimumab may display lower clinical activity than the most frequently used anti-CTLA-4 antibody, ipilimumab^48^. In addition, the use of ICB in earlier stages of disease may have improved activity compared with relapsed/metastatic setting as there is potentially less immune editing and immune evasion in earlier stages of disease. In addition, one limitation of using anti-CTLA-4 for a variety of cancers in the clinic is its toxicity^49^. Systemic delivery of IO agents have been shown to be responsible for severe immune-related adverse events (irAEs), such as colitis, dermatitis, uveitis, and hypophysitis^49^. These adverse events are very toxic, at times irreversible and can even be life-threatening. With this in mind, IT injection may enhance tumor-specific T-cell responses while reducing significant systemic exposure to healthy tissue and off-target toxicities^50,51^. In addition, IT immunotherapy usually causes in situ priming of antitumor immunity, which may allow a patient’s own tumor cells to be used as a therapeutic vaccine^50,51^. In our study, a lower dose of IT anti-PD-1 showed similar therapeutic effects as systemic delivery of a higher dose, and IT anti-CTLA-4 led to complete regression of most 4MOSC1 tumors that are primarily refractory to anti-PD-1. In addition, IT injection led to higher distribution of the antibody in the tumor and cervical lymph nodes, but less in the spleen as a surrogate for distribution in peripheral organs. This suggests that the IT route, which is feasible in HNSCC, may serve as a more effective and less toxic therapeutic strategy for this tumor type, a possibility that may have readily applicable clinical implications, and hence warrant further investigation.

Certainly, some HNSCC tumors have minimal immune infiltration, and may require a multipronged approach to facilitate immune recruitment and activation of the antitumor immune response^52,53^. Other HNSCC lesions are completely refractory to ICB, even if highly immune infiltrated. In this regard, mice implanted with 4MOSC2 failed to respond to anti-PD-1 and anti-CTLA-4 therapy, likely due to the presence of abundant immune suppressive MDSC^30^ in the TIME, which may restrict DC and/or CD8^+^ T-cell function in addition to promoting T-cell exhaustion. Therefore, this 4MOSC model system is ideal for investigating mechanisms of immunotherapy resistance, as well as testing novel multimodal immunotherapies and/or optimization of potential combinations of ICB with chemo- and radiotherapies. Altogether, our findings suggest that our novel syngeneic HNSCC animal models, which strongly mimic tobacco-associated HNSCC and typical clinical situations, may provide experimental tools to investigate interplays between HNSCC and the immune system as well as provide unique opportunities to identify more effective therapeutic strategies for tobacco-associated HNSCC, which are associated with poor prognosis and reduced response to most currently available treatment options.

## Methods

### Reagents

4NQO was purchased from Sigma-Aldrich, dissolved in propylene glycol (Sigma-Aldrich) as a stock solution (4 mg/mL) and stored at 4 °C. PD-1 antibody (clone J43, catalog #BE0033-2), CTLA-4 antibody (clone 9H10, catalog #BP0131), isotype antibody (catalog # BE0091), and CD8 depletion antibody (Clone YTS 169.4, catalog #BE0117) were obtained from Bio X Cell (West Lebanon, NH, USA). Fluorochrome-conjugated antibodies were purchased from BioLegend and BD Biosciences.

### Establishment of cell lines and tissue culture

Female C57Bl/6 mice (4–6 weeks of age and weighing 16–18 g) were purchased from Charles River Laboratories (Worcester, MA, USA). 4NQO was diluted in the drinking water to a final concentration of 50 μg/mL to animals and was changed weekly. After 16 weeks, all animal cages were reverted to regular water until week 22. Animals were euthanized on week 22 for tissue retrieval. Single lesions were dissected, digested, and cells were isolated to establish 4MOSC cell lines.

### DNA sequencing, genomic, and tobacco signature analysis

Raw sequencing data were aligned to the mm10 reference genome using BWA^54^. Somatic mutations were identified by comparing the sequencing data from each cancer sample to the sequencing data from a normal tissue derived from the tail of one of the mice (all mice were genetically identical). To ensure robustness of the results, a consensus variant calling strategy was leveraged in which somatic mutations were identified using three independent bioinformatics tools: Strelka2^55^, Varscan2^56^, and GATK4 Mutect2^57^. Any mutation found in two out of the three variant callers was considered a bona fide somatic mutation. Additional filtering to remove any residual germline contamination was applied and any mutation found in Mouse Genome Project or shared among all four cancers was discarded. Somatic mutational profiles were derived using the immediate sequencing context by evaluating the base 5′ and the base 3′ to each single point mutation. In addition, transcriptional strand bias was evaluated by considering all protein coding genes. Mutational signatures were extracted using our previously developed computational framework SigProfiler^24,25^. SigProfiler can be downloaded freely from: https://www.mathworks.com/matlabcentral/fileexchange/38724-sigprofiler.

Gene mutation analyses were performed comparing our four syngeneic cells to a HNSCC provisional dataset containing 243 HPV− tumor samples from the publicly available consortium, TCGA^40^. Mutational plots of p53 mutations observed in characterized HNSCC samples from TCGA and four of our syngeneic cell lines were summarized using the “lolipop” mutation diagram generator^58^.

### In vivo mouse experiments and analysis

All the animal studies using HNSCC tumor xenografts and oral carcinogenesis studies were approved by the Institutional Animal Care and Use Committee (IACUC) of University of California, San Diego, with protocol ASP #S15195. Mice at Moores Cancer Center, UCSD are housed in micro-isolator and individually ventilated cages supplied with acidified water and fed 5053 Irradiated Picolab Rodent Diet 20 from lab diet. Temperature for laboratory mice in our facilitiy is mandated to be between 65–75 °F (~18–23 °C) with 40–60% humidity. All animal manipulation activities are conducted in laminar flow hoods. All personnel are required to wear scrubs and/or lab coat, mask, hair net, dedicated shoes, and disposable gloves upon entering the animal rooms. 4MOSC1 and 4MOSC2 cells were transplanted (1 million per mouse) into the tongue of female C57Bl/6 mice (4–6 weeks of age and weighing 16–18 g). When tumors were formed (on day 5–6), the mice were first randomized into groups. For drug treatment, the mice were treated by either intraperitoneal (IP) or IT injection with isotype control antibody, PD-1 antibody, or CTLA-4 antibody (IP 10 mg/kg, IT 5 mg/kg, three times a week) for 3 weeks. The mice were then euthanized after the completion of the treatment (or when control-treated mice succumbed to tumor burdens, as determined by the ASP guidelines) and tumors were dissected for flow cytometric analysis or histologic and immunohistochemical evaluation.

For *Foxp3*^DTR^ mice, we use *Foxp3*-GFP-DTR mice (C57BL/6-Tg(Foxp3-DTR/EGFP); from JAX in C57BL/6 background) (6–8 weeks of age and weighing 18–22 g). To deplete Tregs, mice were injected IP with 500 ng of diphtheria toxin (DT; Sigma-Aldrich), diluted in PBS.

### Chemokine expression profile

Tongue tumors were dissected and lysed in RIPA lysis buffer supplemented with protease and phosphatase inhibitors^59^. Samples were run on the Mouse Chemokine Array 44-Plex (EVE Technologies, Canada).

### Immunofluorescence and image quantification

Briefly, tissues (tongue, cervical lymph nodes, and spleen) were harvested, fixed, and paraffin embedded. Slides were stained for CK5 (Fitzgerald, 20R-CP003) (1:500) and CD8 (abcam, ab22378) (1:400) antibodies. Quantification of immune infiltration was done using QuPath, an open source software for digital pathology image analysis^60^. For the quantification, at least three regions of interest (ROI) were selected for each condition and the percentage of positive cells for the CD8 marker was calculated. In order to quantify the immune-fluorescent-stained Foxp3 and CD8 positive cells in the *Foxp3*^DTR^ mice, we quantified the number of positive cells in each ROI. CD8 antibody (catalog # ab22378) (1:400) was purchased from Abcam (Cambridge, United Kingdom) and FoxP3 antibody (catalog #D608R) (1:200) was purchased from Cell Signaling Technology (Danvers, MA).

### TIL isolation and flow cytometry

Tumors were dissected, minced, and resuspended in complete media (DMEM with 10% FBS and 1% antibiotics) supplemented with Collagenase-D (1 mg/mL; Roche) and incubated at 37 °C for 30 min with shaking to form a single-cell suspension. Tissue suspensions were washed with fresh media and passed through a 100-µm strainer. Samples were washed with PBS and immediately processed for live/dead cell discrimination using BD Horizon^TM^ Fixable Viability Stain 510. Cell surface staining was done for 30 min at 4 °C with the following antibodies (all from BioLegend, San Diego, CA): CD45 (30-F11) (1:100), CD3 (145-2C11) (1:400), CD8a (53-6.7) (1:100), CD4 (RM4-4) (1:400), NK1.1 (PK136) (1:400), CD24 (M1/69) (1:100), MHCII (M5/114.15.2) (1:800), Ly6-G (1A8) (1:400), Ly6-C (HK1.4) (1:100), F4/80 (T45-2342) (1:100), CD103 (2E7) (1:100), CD11b (M1/70) (1:200), CD11c (HL3) (1:100), PD-1 (29 F.1A12) (1:100), TIM-3 (B8.2C12) (1:100), and CD44 (IM7) (1:100). Intracellular staining for inhibitory receptors LAG-3 and CTLA-4 was done using the BD Cytofix/Cytoperm kit and stained with the LAG-3 (C9B7W) (1:100) and CTLA-4 (UC10-4B9) (1:100) antibodies. Intracellular staining for FOXP3 was performed using the eBioscience FOXP3/Transcription factor buffer set from invitrogen and stained with the FOXP3 (MF23) antibody. All flow cytometry data acquisition was done using BD LSRFortessa and analyzed using FlowJo software. TIL count was determined using BD Trucount^TM^ tubes. Immune cells were identified by the following characteristics: cytotoxic T cells (CD45^+^Thy1.2^+^CD8^+^), helper T cells (CD45^+^Thy1.2^+^CD4^+^), Treg (CD45^+^Thy1.2^+^CD4^+^FoxP3^+^), NK cells (CD45^+^Thy1.2^−^NK1.1^+^), macrophages (CD45^+^Thy1.2^−^NK1.1^−^CD11b^+^CD11c^−^LY6C^low^LY6G^low^CD24^+^F4/80^+^), PMN-MDSCs (CD45^+^Thy1.2^−^NK1.1^−^CD11b^+^CD11c^−^LY6C^low^LY6G^+^), and M-MDSCs (CD45^+^Thy1.2^−^NK1.1^−^CD11b^+^CD11c^−^LY6C^+^LY6G^low^). A representative flow cytometry gating strategy is depicted in Supplementary Fig. 12.

### Antigen specific T-cell cytotoxicity assay

4MOSC1 tumors were mechanically and enzymatically digested as described above and tumor-derived T cells were isolated by the Murine CD8a^+^ T Cell Isolation Kit from Miltenyi Biotec (Bergisch Gladbach, Germany). 4MOSC1 cells were plated in keratinocyte media in the 24-well µ-plate from ibidi (Grafelfing, Germany) and when cells grew to 60% confluency, T cells were added at a 1:10 cancer cell to T cells ratio. The viability dye, DRAQ7, was added in the culture medium to discriminate cancer cell killing by T cells, and T cells were labeled with Vybrant Dil Cell-Labeling Solution from Invitrogen (Carlsbad, CA). Overnight live-imaging was captured in real time by the Zeiss LSM 880 confocal with Airyscan FAST.

### NanoString analyses

RNA was isolated from tumor samples using the RNeasy Micro Kit (Qiagen 74004). Hybridization of samples was done according to the NanoString Hybridization Protocol for nCounter XT CodeSet Gene Expression Assays. Samples were run on the nCounter SPRINT Profiler with the nCounter PanCancer Mouse Immune Profiling gene expression platform. Analysis of gene expression was done using the Advanced Analysis module on the nSolver software.

*Statistics and reproducibility:* Statistical data analyses, variation estimation, and validation of test assumptions were carried out with GraphPad Prism version 7 statistical analysis program (GraphPad Software, San Diego, CA). All analyses were performed in triplicate or greater and the means obtained were used for independent *t*-tests, ANOVA, or longitudinal data analysis method. The asterisks denote statistical significance (nonsignificant or ns, *P* > 0.05; *P < 0.05; **P < 0.01; and ***P < 0.001). All the data are reported as mean ± standard error of the mean (S.E.M.). For all experiments, each experiment was repeated independently with similar results for at least three times.

### Reporting summary

Further information on research design is available in the Nature Research Reporting Summary linked to this article.

## Supplementary information

Supplementary Information

Description of Additional Supplementary Files

Supplementary Data 1

Supplementary Data 2

Supplementary Data 3

Supplementary Data 4

Reporting Summary

## Data Availability

The whole exome sequencing data of murine 4NQO-induced syngeneic cell lines have been deposited in the NCBI Sequence Read Archive (SRA) database under the accession code PRJNA575532. The whole exome sequencing data referenced during the study are available in a public repository from the NCBI SRA website. The source data underlying Fig. 1b–e and Supplementary Fig. 1 are provided as Supplementary Data 1–4 in microsoft excel format. All the other data supporting the findings of this study are available within the article and its Supplementary Information files and from the corresponding author upon reasonable request. A reporting summary for this article is available as a Supplementary Information file.
