# Supplementary material for: Syngeneic animal models of tobacco-associated oral cancer reveal the activity of in situ anti-CTLA-4
Source: Nat Commun. 2019 Dec 5;10:5546. doi: 10.1038/s41467-019-13471-0 (PMC6895221; doi:10.1038/s41467-019-13471-0)
Supplement: Supplementary file 1 — Supplementary Information [file 41467_2019_13471_MOESM1_ESM.pdf]

Supplementary information for:

Syngeneic animal models of tobacco-associated oral cancer reveals the activity of *in situ* anti-CTLA-4

Wang *et al*.,

Supplementary Figure 1

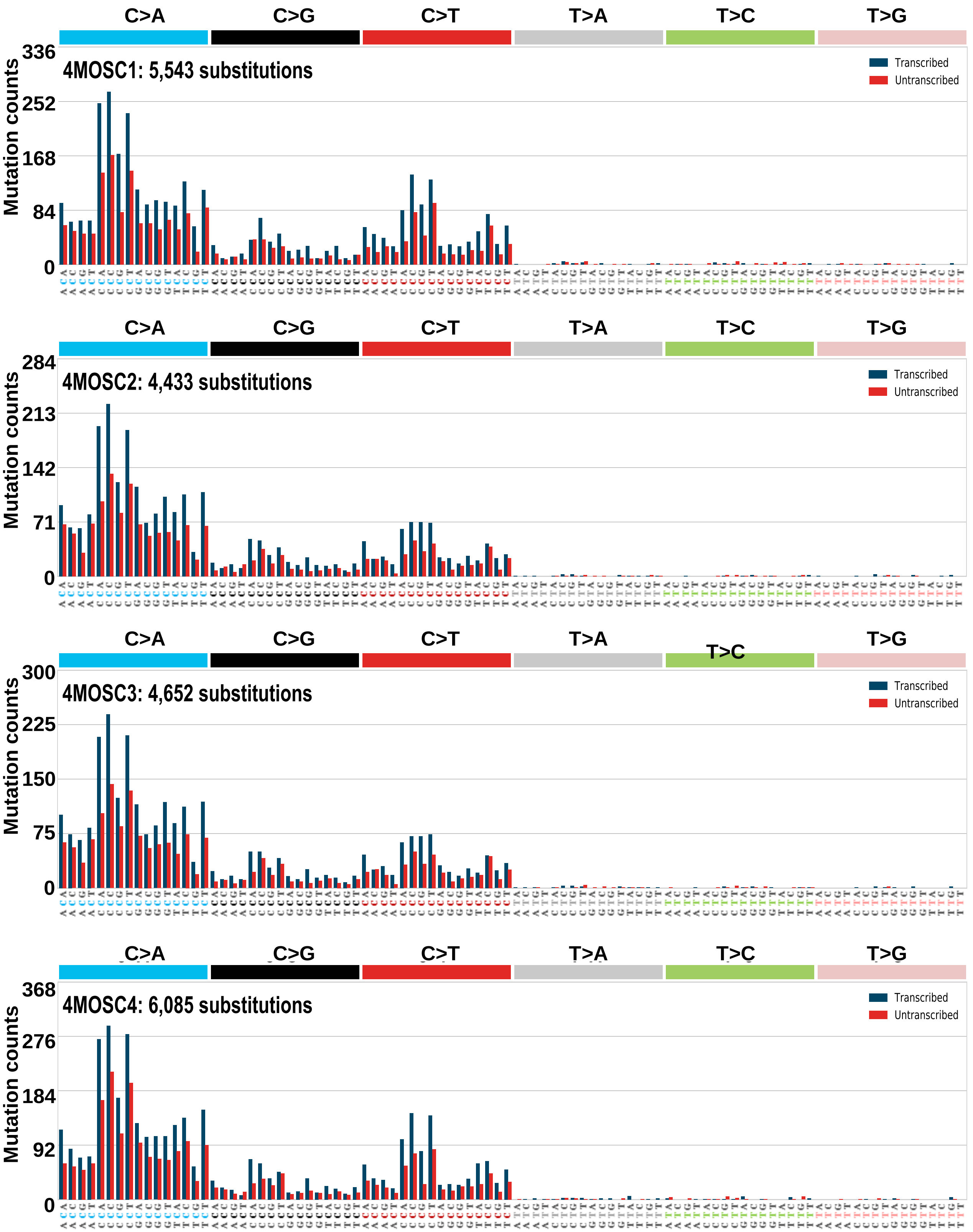

Supplementary Figure 1.Transcriptional Bias for each individual 4MOSC cell lines. The somatic mutational profiles of the four 4MOSCs were correlated to (Pearson correlation > 0.93), known mutational signatures in human cancer. The pattern of Signature 4 extracted from cancers associated with tobacco smoking was marked as dark blue columns.

Supplementary Figure 2

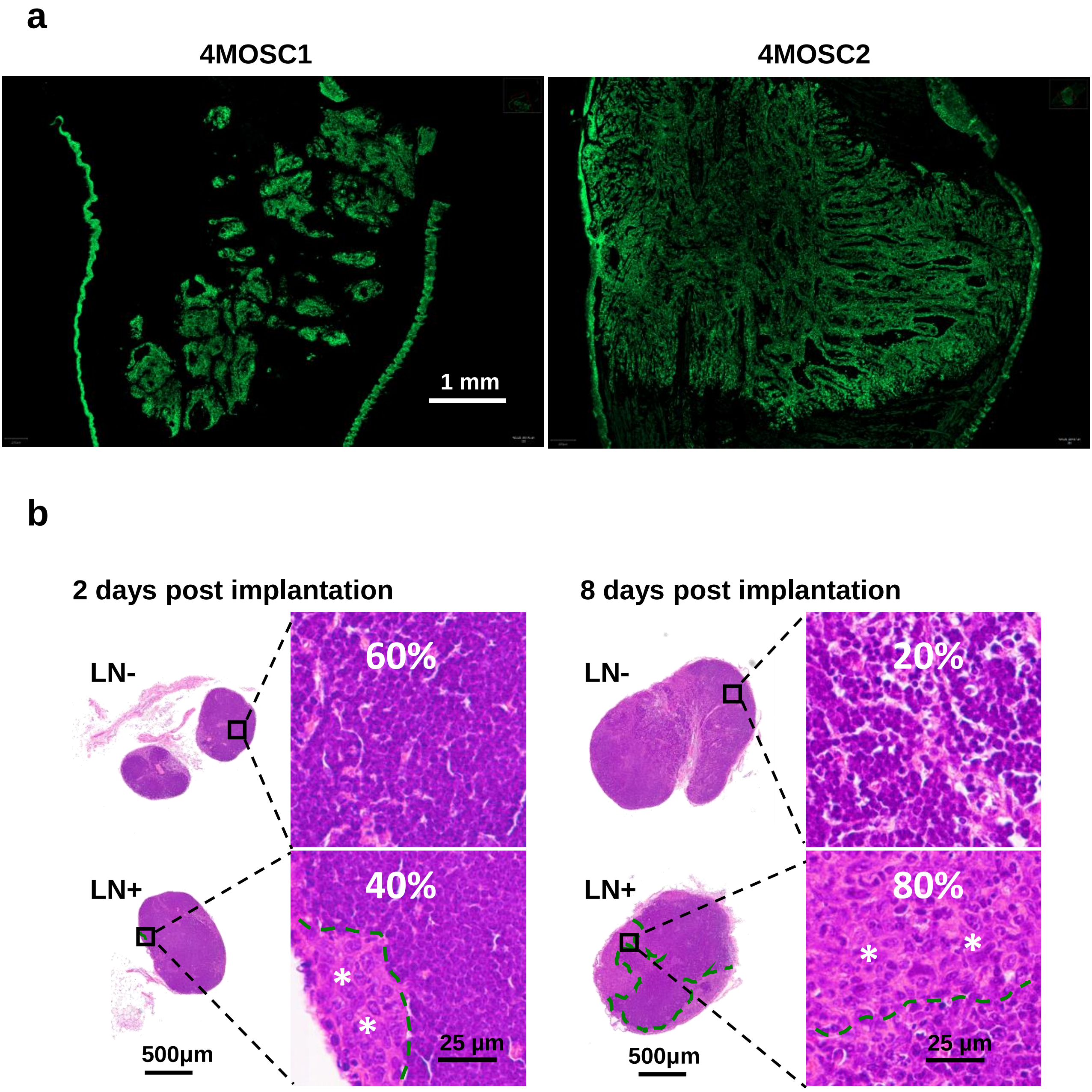

Supplementary Figure 2. Squamous cell character. (a) Representative pictures of whole tongue tumors stained to show expression of cytokeratin 5 (CK5, green); left, 4MOSC1; right, 4MOSC2 (n = 3 mice per group). **(b)** Metastatic growth of 4MOSC2 cells into the lymph node. C57Bl/6 mice were implanted with 1×10^6 of 4MOSC2 cells into the tongue. On 2 and 8 days post-implantation, cervical lymph nodes from each mouse were harvested and evaluated by H&E staining. Left, representative H&E stain of a non-metastatic (top) and a metastatic (bottom) cervical lymph node. Right, images at high magnification depict the histologic features of representative area from each individual cervical lymph node. Metastatic area is depicted with a green dotted line, with the tumor cells marked by * (n = 5 mice per group).

Supplementary Figure 3

Fold difference
1
1 to 2
2.1 to 4
4.1 to 10
> 10

	Relative value		Concentration (pg/ml)		
4MOSC	1	2	4MOSC1	4MOSC2	Sig.
CCL11	2.9		292±10	100±10	***
CCL12	1.2		314±63	268±30	ns
CCL17	7.1		361±255	51±10	ns
CCL19		2.4	48.2±6.6	118±13	**
CCL2	3.1		1339±98	430±54	***
CCL20	1.7		1.2±0.2	0.7±0.1	*
CCL21	1.7		1205±114	719±41	**
CCL22	4.4		73.6±6.2	16.8±0.9	***
CCL3	1.6		312±17	198±10	***
CCL4	2.9		295±26	102±5	***
CCL5	4.9		84.8±6.1	17.4±2.6	***
CX3CL1	1.4		132±14	96.9±6.3	ns
CXCL1		1.7	654±110	1137±75	**
CXCL10		4.0	694±82	2801±1156	ns
CXCL2		1.9	4398±835	8171±234	**
CXCL5		8.5	361±21	3052±375	***
EPO	1.5		2.4±0.2	1.6±0.1	**
G-CSF		13.7	355±73	4877±68	***
GM-CSF		13.6	52±14	704±51	***
IFNβ1		2.5	80.6±7.8	204±99	ns
IFNγ	1.6		4.8±1.0	3.1±0.4	ns
IL-10		1.3	2.7±0.4	3.6±0.3	ns
IL-11	1.9		460±138	244±51	ns
IL-12 (p40)	1.3		2.8±0.3	2.2±0.3	ns
IL-12 (p70)	1.2		4.2±0.8	3.6±0.2	ns
IL-13	1.2		4.7±0.2	3.9±0.3	ns
IL-15	1.1		6.0±0.3	5.7±0.4	ns
IL-16	1.5		795±52	543±63	*
IL-17	1.8		1.8±0.2	1.0±0.1	**
IL-1a		1.8	216±13	383±33	**
IL-1b		1.3	48.6±8.9	65.6±5.2	ns
IL-2			6.5±0.4	6.5±0.7	ns
IL-20	1.1		7.5±0.3	7.1±0.3	ns
IL-3	2.8		4.0±2.2	1.4±0.2	ns
IL-4	10.3		3.1±0.6	0.3±0.1	***
IL-5	6.2		8.1±1.3	1.3±0.2	***
IL-6	1.8		127±32	69.7±8.8	ns
IL-7		2.1	2.6±0.2	5.4±0.1	***
IL9	1.2		11.1±2.4	9.4±2.8	ns
LIF	1.6		88.9±7.2	54.9±5.3	**
M-CSF	1.2		28.2±3.2	24.5±3.0	ns
TIMP-1	1.8		6242±927	3510±757	ns
TNFα	1.3		13.9±1.4	10.6±1.1	ns
VEGF		26.8	1.4±0.2	37.5±6.5	***

Supplementary Figure 3. Chemokine expression profile of the 4MOSC tumors. C57Bl/6 mice were implanted with 1x10^6 of either 4MOSC1 or 4MOSC2 cells into the tongue. Eleven days post-implantation, tongue tumors were dissected and lysed. Tumor lysates were normalized to 1 mg/mL and analyzed to quantify concentrations of multiple chemokines, cytokines, and growth factors. Left, relative values of each chemokine in 4MOSC1 and 4MOSC2; fold differences were calculated by dividing the tumor with the higher concentration by the tumor with the lower concentration, and the tumor with the lower concentration was defined as 1-fold. Right, absolute concentration of each chemokine in 4MOSC1 and 4MOSC2 (n = 5 mice per group; not significant or ns, $p > 0.05$; *, $p < 0.05$; **, $p < 0.01$; and ***, $p < 0.001$ when comparing 4MOSC1 with 4MOSC2 with two sided Student's *t*-test; data are represented as mean± SEM).

Supplementary Figure 4

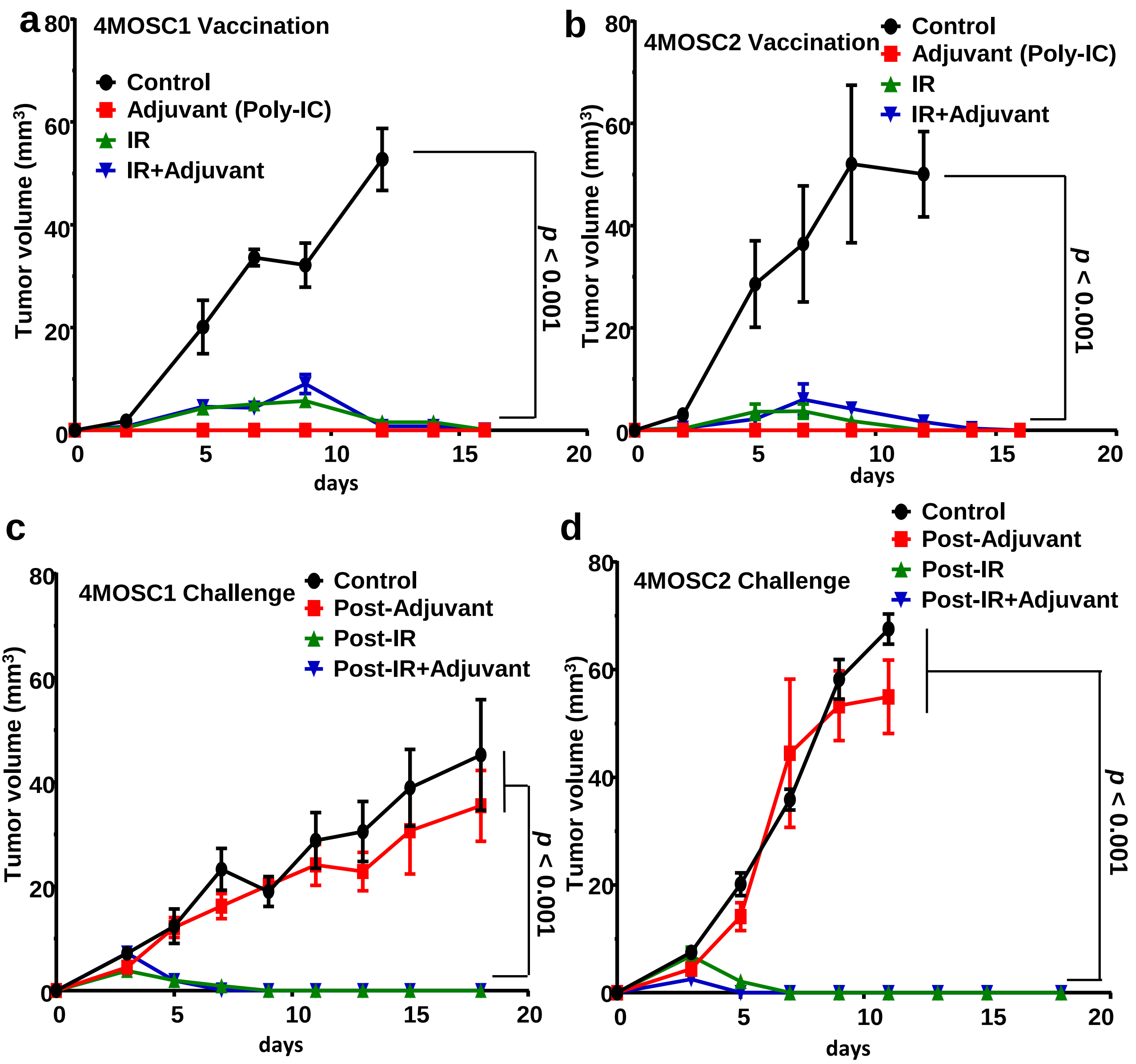

Supplementary Figure 4. Immunogenicity of 4MOSCs. (a-b) Memory immune responses induced by vaccination with irradiated 4MOSC cells. 4MOSC1 or 4MOSC2 cells were irradiated with 45 Gy and 1x10^6 cell were injected into the tongue of C57Bl/6 mice, with (green) or without (blue) polyinosinic-polycytidylic acid (poly IC). Mice injected with non-irradiated 4MOSC cells (black) or mice only treated by poly IC (red) were used as controls. The average tumor volume of each group is shown (n = 5 mice per group, the tumor growth curves were compared by the longitudinal data analysis method; data are represented as mean± SEM). **(c-d)** Vaccinated mice (green and blue) were re-challenged with 1x10^6 live 4MOSC cells 6 weeks after. Naïve mice (black) and mice post poly IC treatment (red) were used as controls. The average tumor volume of each group is shown (n = 5 mice per group, the tumor growth curves were compared by the longitudinal data analysis method; data are represented as mean± SEM).

Supplementary Figure 5

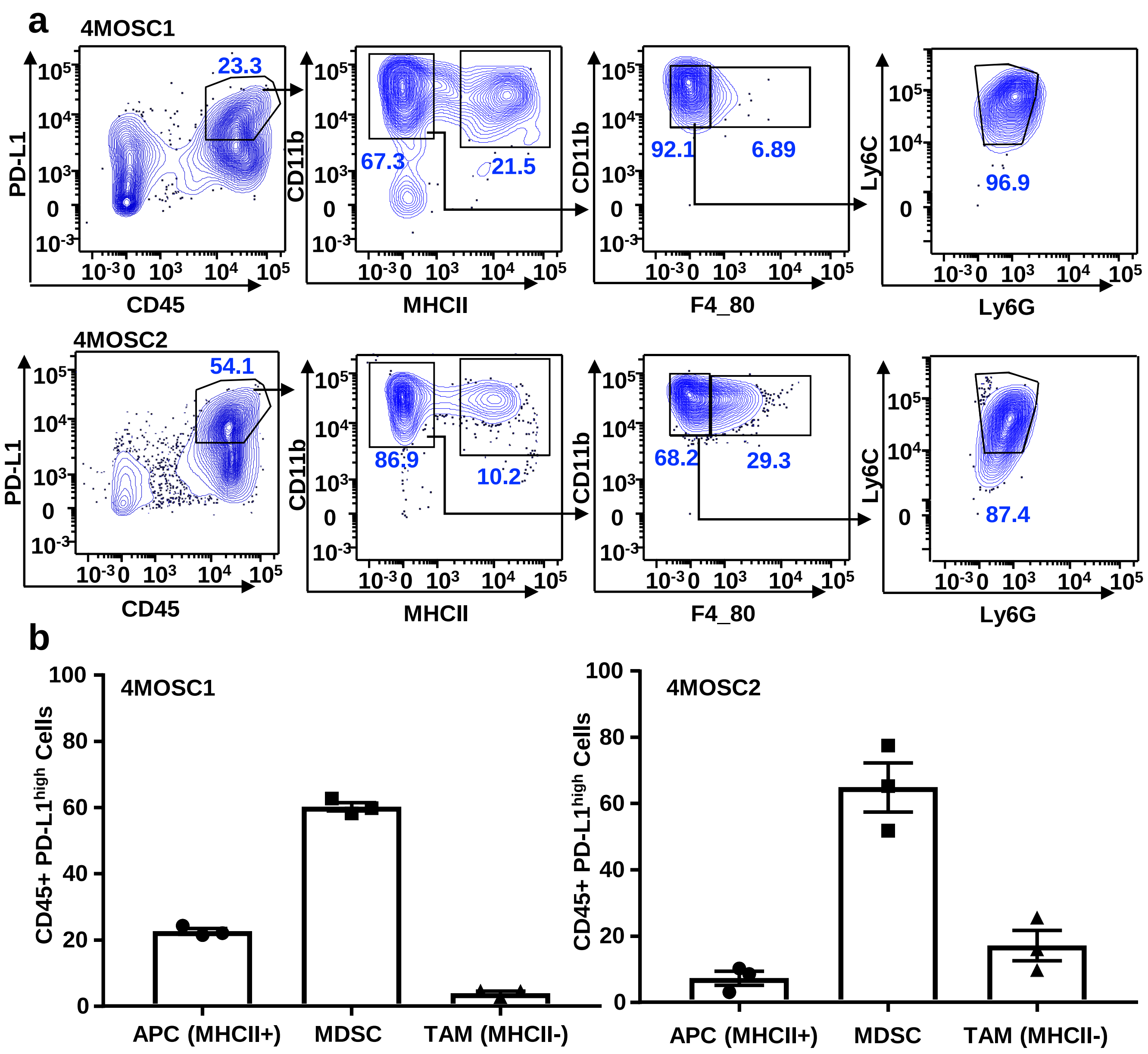

Supplementary Figure 5. PD-L1 is expressed on tumor and tumor-infiltrating myeloid immune cells. Frequency of live 4MOSC1 or 4MOSC2 tumors expressing PD-L1. **(a)** Shown are representative flow cytometry plots of PD-L1 expression on 4MOSC cells, CD45+ immune cells, $MHCII^{+}$ antigen presenting cells (APC), $F4/80^{+}$ $MHCII^{-}$ tumor-associated macrophages (TAMs), and $Ly6C^{+}Ly6G^{+}$ MDSCs. **(b)** The averaged frequency of each immune cell population expressing $PD\text{-}L1^{high}$ in 4MOSC1 and 4MOSC2 is shown (n = 3 mice per group; data are represented as mean± SEM).

Supplementary Figure 6

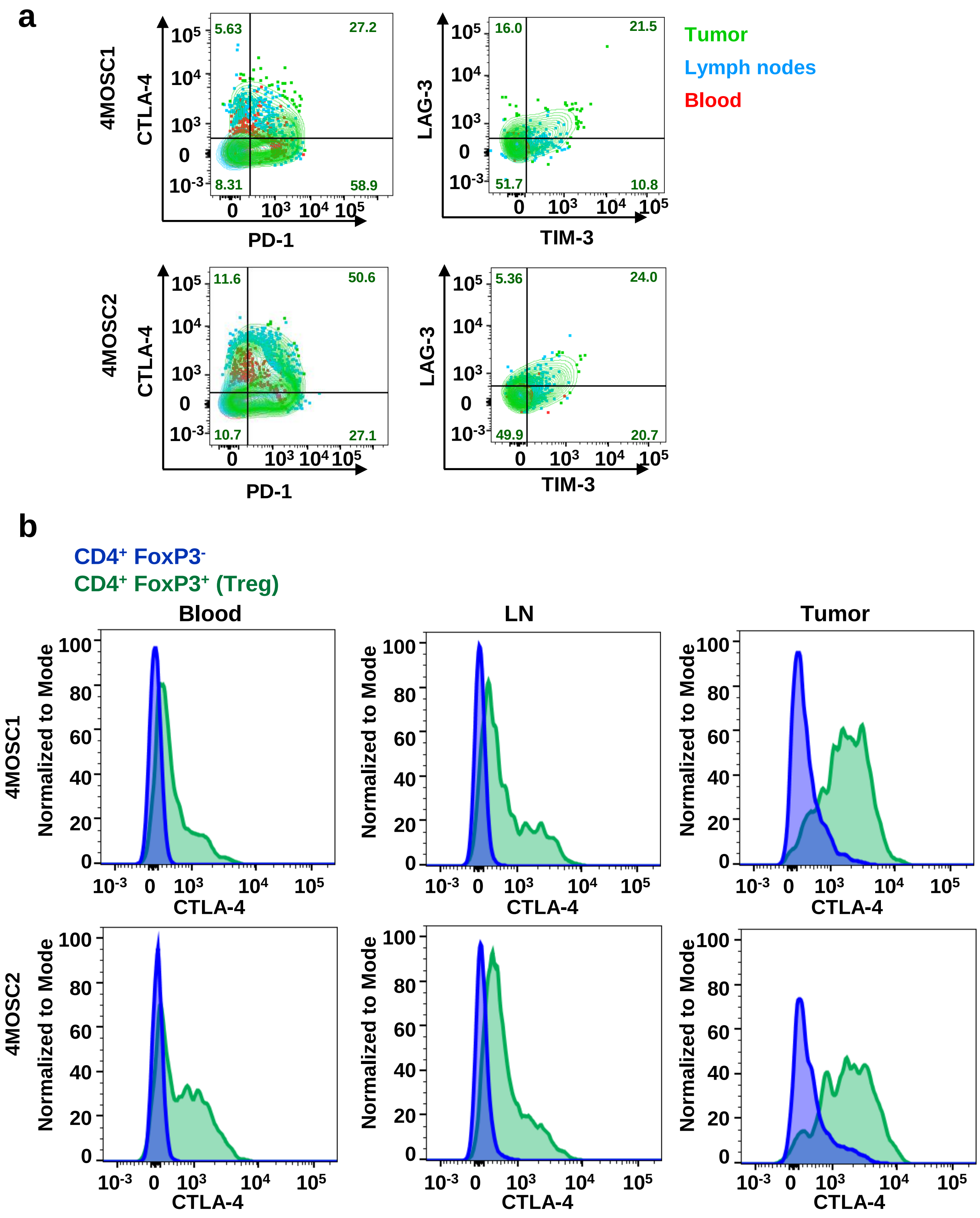

Supplementary Figure 6. Characterization of inhibitory receptor expression on tumor-infiltrating CD4 T cells in 4MOSCs. Frequency of live 4MOSC1 or 4MOSC2 tumors expressing inhibitory receptors. **(a)** Shown are representative flow cytometry plots of the frequency of $CD4^+$ cells expressing inhibitory receptors PD-1, CTLA-4, LAG-3 and TIM-3 (n = 4 mice per group). Contour plots of lymphocytes from tumor (green), and corresponding cervical lymph nodes (blue), and blood (red) are overlaid and the frequencies of tumor $CD4^+$ T cells expressing each inhibitory receptor are shown. **(b)** The expression of CTLA-4 on CD4 T cells, Tregs ($CD4^+FoxP3^+$) or non-Tregs ($CD4^+FoxP3^-$) are represented by overlaid histograms in blood, LN, and tumor (n = 4 mice per group).

Supplementary Figure 7

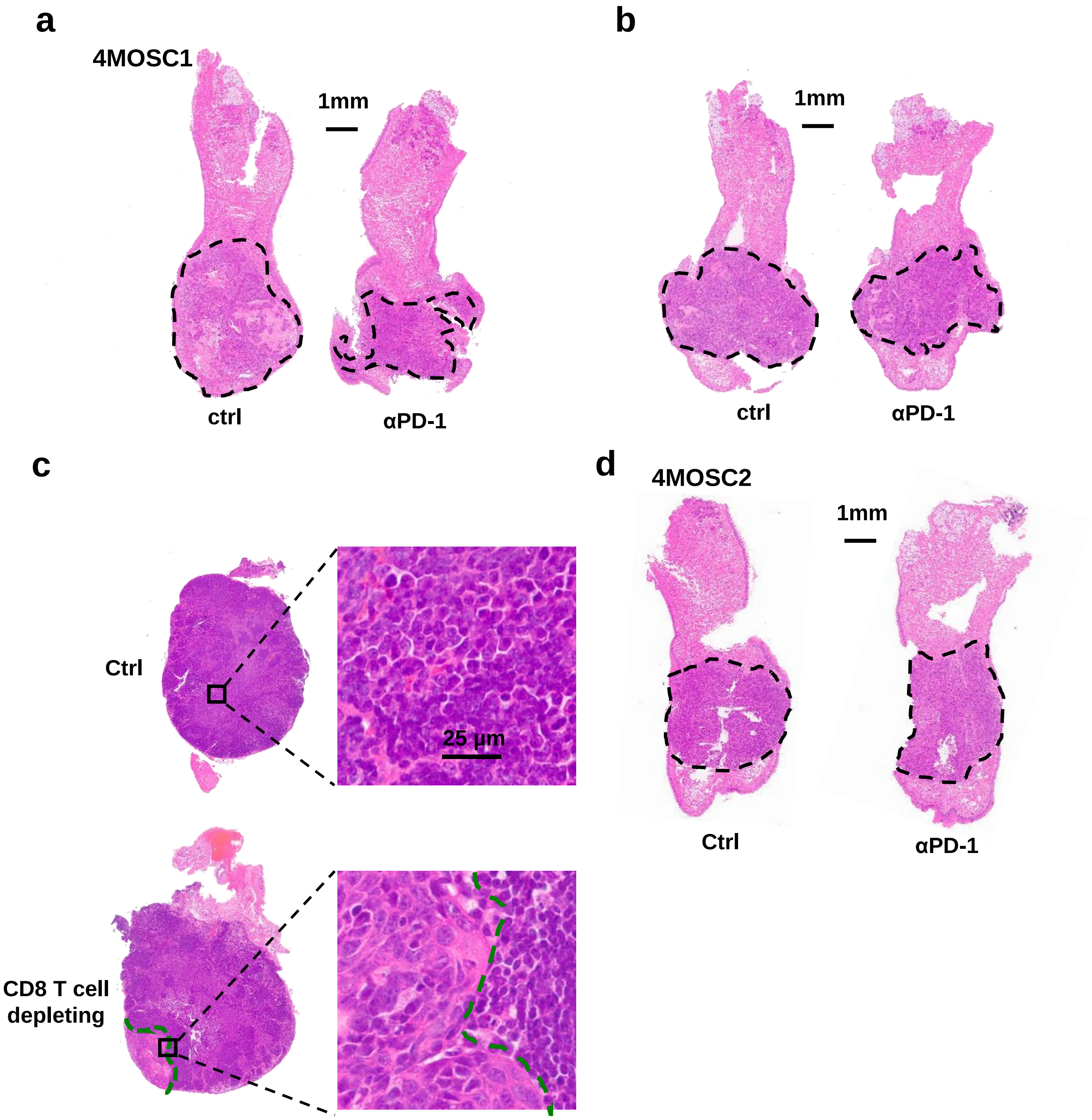

Supplementary Figure 7. Histopathological analysis of tongues and cervical lymph nodes from 4MOSC1 or 4MOSC2 tumor-bearing mice. (a-b) Representative H&E stains of mouse tumors from the experiment in panel 3a (n = 10 mice per group) and 3e(n = 5 mice per group). The H&E stained tissue section of an HNSCC tumor is depicted with a dotted line. **(c)** Top panel, representative H&E stain of a non-metastatic cervical lymph node from mice with 4MOSC1 tumors. Bottom panel, representative H&E stain of a metastatic cervical lymph node from mice with 4MOSC1 tumors after treatment with CD8 T cell-depleting antibody. Metastatic growth of 4MOSC1 cells into the lymph node is depicted with a dotted line in the left area. ((n = 5 mice per group)) **(d)** Representative H&E stains of mouse tumors from the experiment in panel 3g. The H&E stained tissue section of an HNSCC tumor is depicted with a dotted line (n = 5 mice per group).

Supplementary Figure 8

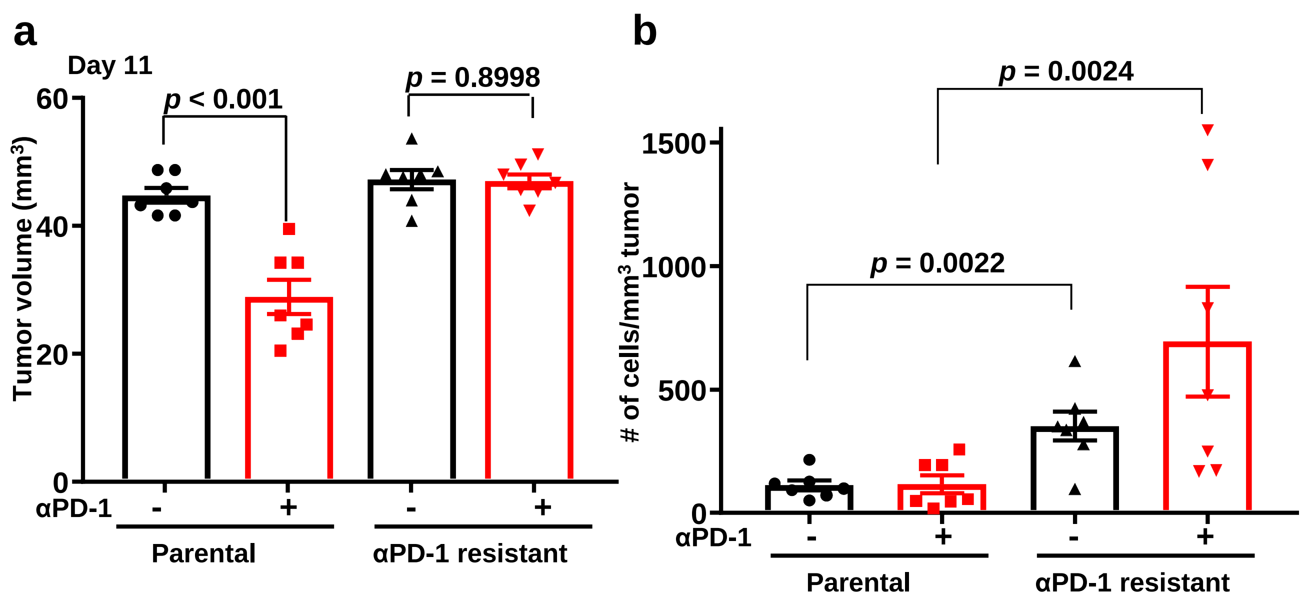

c

CD8 — Parental

αPD-1	-	+	Sig.
CTLA-4⁺	1.00	1.09	ns
LAG-3⁺	1.00	1.19	ns
PD-1⁺	1.00	0.60	**
TIM-3⁺	1.00	1.00	ns

CD8 — αPD-1 resistant

αPD-1	-	+	Sig.
CTLA-4⁺	1.00	1.18	**
LAG-3⁺	1.00	1.83	***
PD-1⁺	1.00	0.69	**
TIM-3⁺	1.00	1.37	***

CD4 — Parental

αPD-1	-	+	Sig.
CTLA-4⁺	1.00	1.10	ns
LAG-3⁺	1.00	1.78	**
PD-1⁺	1.00	0.57	***
TIM-3⁺	1.00	0.96	ns

CD4 — αPD-1 resistant

αPD-1	-	+	Sig.
CTLA-4⁺	1.00	1.06	ns
LAG-3⁺	1.00	2.08	**
PD-1⁺	1.00	0.56	***
TIM-3⁺	1.00	1.07	ns

Fold: 0.5 — 1 — 2

Supplementary Figure 8. Difference of immune infiltration and immune checkpoints in parental 4MOSC1 and αPD-1-resistant 4MOSC1. Anti-PD-1-resistant 4MOSC1 cell lines were established by first isolating cells from anti-PD-1-treated mice showing no response and re-injecting into C57Bl/6 mice. This process was repeated for a total of 3 rounds to generate the resistant cell line. C57Bl/6 mice were implanted with $1x10^6$ parental or anti-PD1-resistant 4MOSC1cells. After the tumors reached ~30 mm^3, mice were treated IP with 10mg/kg of isotype control or 10mg/kg of anti-PD-1 every other day for 3 treatments total. **(a)** Shown is the average volume of each tumor at the endpoint of the experiment with error bars representing standard error (n = 7 mice per group; two sided Student's *t*-test; data are represented as mean ± SEM). **(b)** Quantification of tumor-infiltrating PMN-MDSCs ($Ly6G^{hi}$) was performed by flow cytometry (n = 7 mice per group; two sided Student's *t*-test; data are represented as mean ± SEM). **(c)** Shown is the average fold change of the frequency of tumor-infiltrating CD4 and CD8 T cells expressing inhibitory receptors from anti-PD-1-treated parental and resistant 4MOSC1 (n = 7 mice per group; not significant or ns, $p > 0.05$; *, $p < 0.05$; and ***, $p < 0.001$, two sided Student's *t*-test).

Supplementary Figure 9

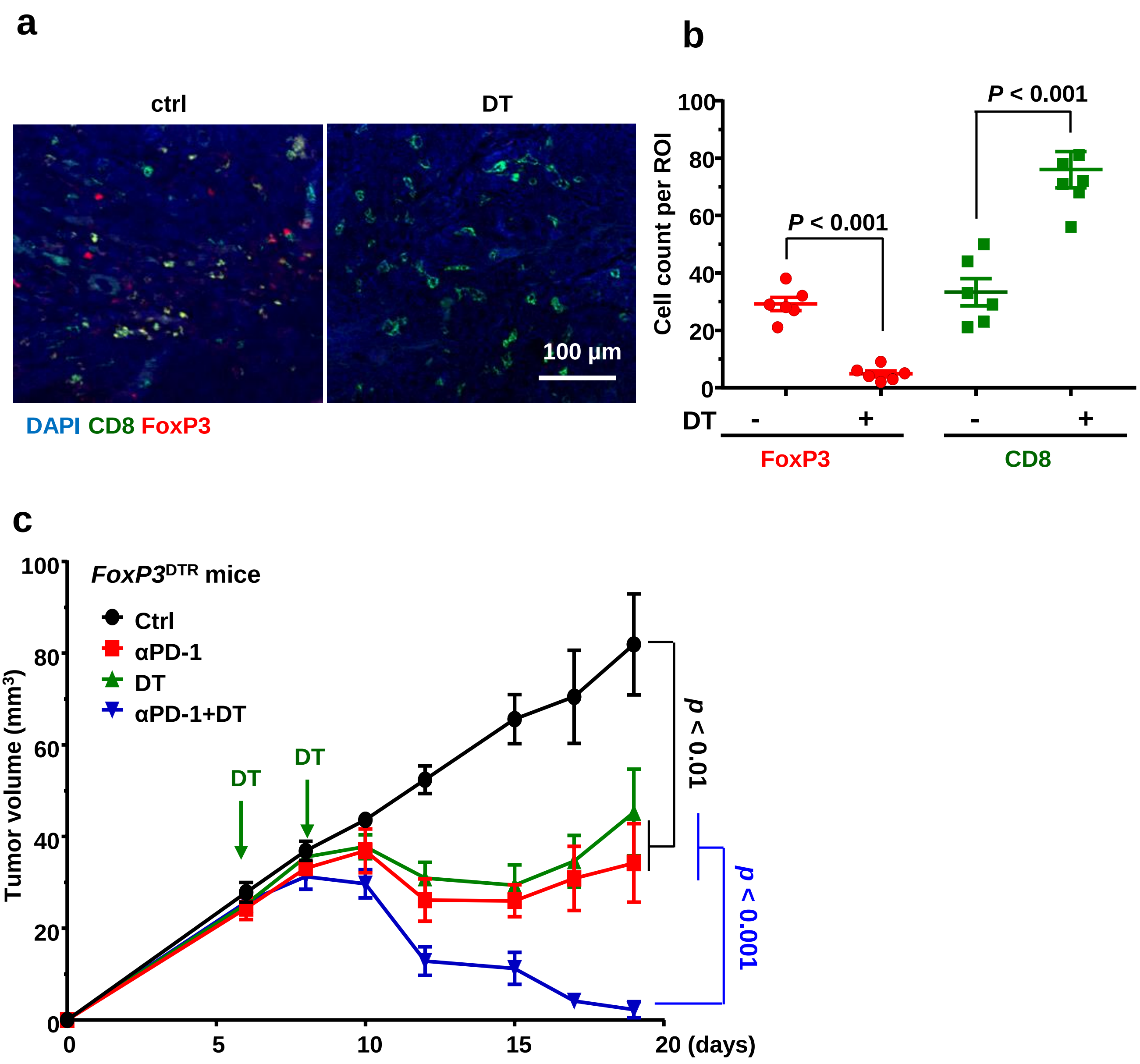

Supplementary Figure 9. Treg-mediated suppression of anti-PD-1 activity in 4MOSC1 tumors in *FoxP3*^{DTR} mice. (a) *FoxP3*^{DTR} mice were implanted with 1x10^{6} of 4MOSC1 cells into the tongue, and when they reached approximately 30 mm^{3}, mice were treated IP with PBS or diphtheria toxin (DT). Immunofluorescent staining of isolated tumors with FoxP3 and CD8 confirm transient elimination of Tregs with DT and increase in CD8 T cells. **(b)** Shown is the quantification of the FoxP3 and CD8 positive cells by 3 regions of interests (ROI) per mouse, quantified by Qupath software for mice untreated and treated with DT. (n = 6 mice per group; two sided Student's *t*-test; data are represented as mean± SEM). **(c)** *FoxP3*^{DTR} mice with 4MOSC1 tongue tumors were treated IP with 10mg/kg of isotype control (black), 10mg/kg of anti-PD-1 (red), diphtheria toxin (green) or both (blue). (n = 5 mice per group; the tumor growth curves were compared by the longitudinal data analysis method; data are represented as mean± SEM).

Supplementary Figure 10

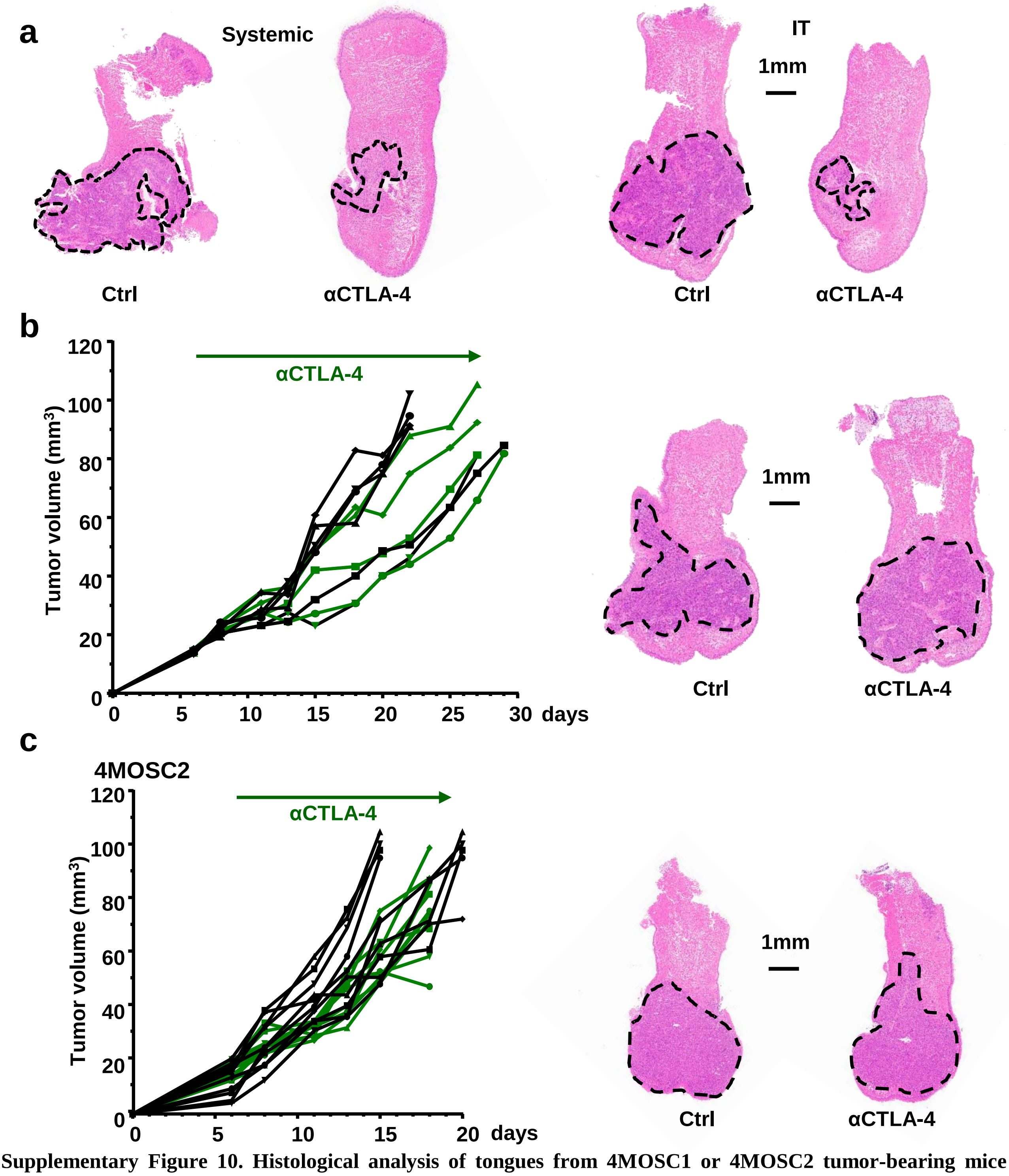

Supplementary Figure 10. Histological analysis of tongues from 4MOSC1 or 4MOSC2 tumor-bearing mice treated with anti-CTLA-4. (a) Representative H&E stains of mouse tumors from the experiment in panel 4a. The H&E stained tissue section of an HNSCC tumor is depicted with a dotted line (n = 10 mice per group). **(b)** Left panel, anti-CTLA-4 dependency on CD8 T cells. C57Bl/6 mice were treated with a CD8 T cell-depletion antibody, and transplanted with $1x10^6$ 4MOSC1 cells into the tongue. After the tumors reached ~30 mm^3, mice were treated IT with 5 mg/kg of isotype control (black) or anti-CTLA-4 (green) (n = 5 per group). Individual growth curves of 4MOSC1 tumor-bearing mice plotting primary tumor growth were recorded. Right panel, representative H&E of mouse tumors from the experiment in left panel. The H&E stained tissue section of an HNSCC tumor is depicted with a dotted line. **(c)** Left panel, antitumor efficacy of anti-CTLA-4 for mice with 4MOSC2 tumors. C57Bl/6 mice were transplanted with $1x10^6$ 4MOSC2 cells into the tongue. After the tumors reached ~30 mm^3, mice were treated IT with 5 mg/kg of isotype control (black) or anti-CTLA-4 (green) (n = 10 mice per group). Individual growth curves of 4MOSC2 tumor-bearing mice plotting primary tumor growth were recorded. Right panel, representative H&E of mouse tumors from the experiment in left panel. The H&E stained tissue section of an HNSCC tumor is depicted with a dotted line.

Supplementary Figure 11

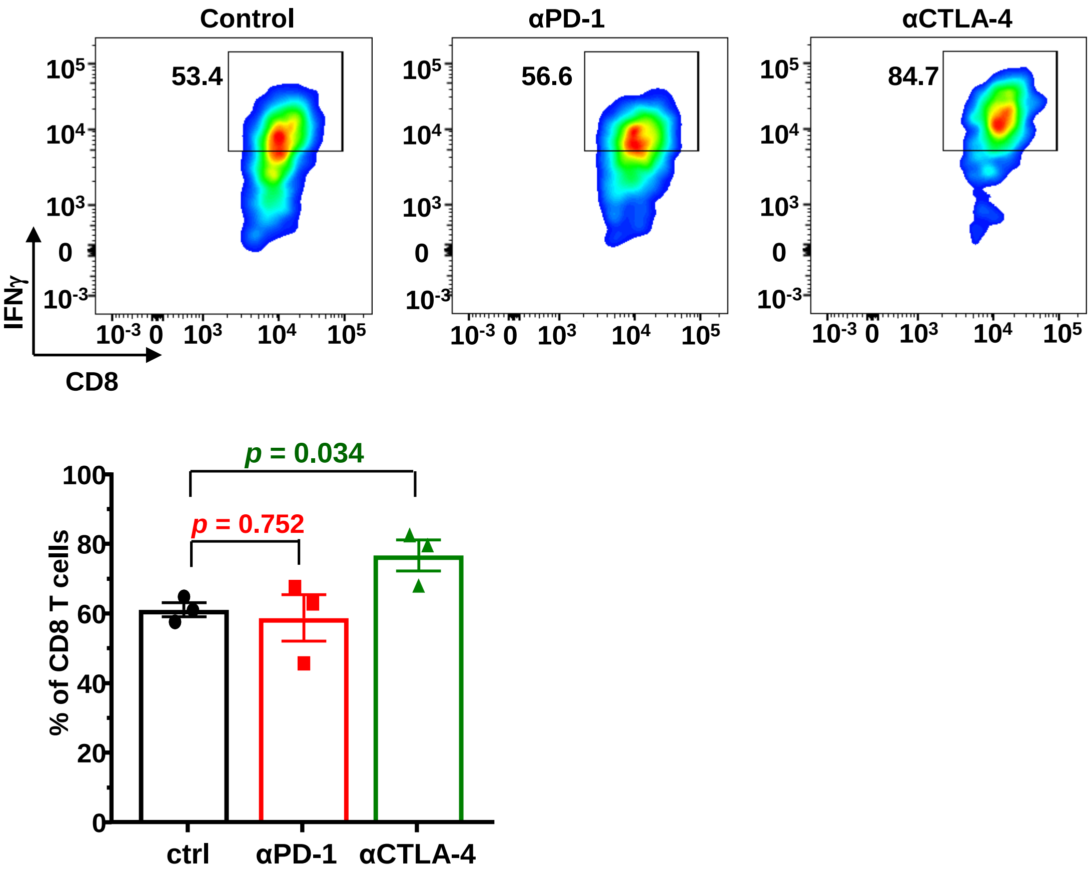

Supplementary Figure 11. Increased expression of IFNg in CD8 T cells by anti-CTLA-4 treatment. Frequency of $CD45^+$, $Thy1.2^+$, $CD8^+$ expressing IFNγ. Top panel, a representative flow cytometry plot from one mouse showing the frequency of $IFN\gamma^+$ out of $CD8^+$ cells is shown. Bottom panel, the frequency of $IFN\gamma^+CD8^+$ cells was quantified following treatment with anti-PD-1 or anti-CTLA-4 (n = 3 mice per group; two sided Student's *t*-test; data are represented as mean± SEM).

Supplementary Figure 12

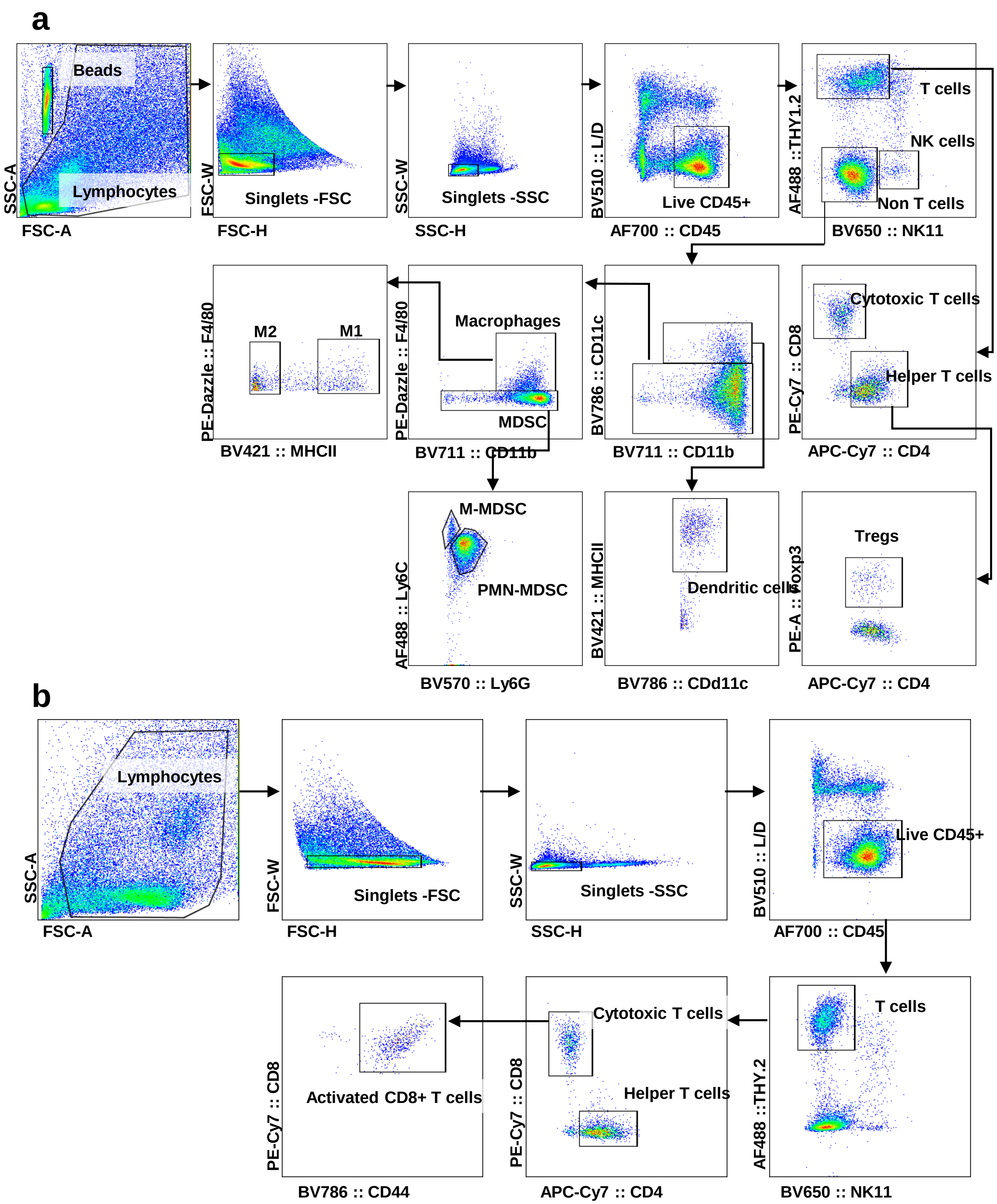

Supplementary Figure 12. Representative flow cytometry gating strategies. Representative flow cytometry plots to gate **(a)** tumor-infiltrating immune cells used to quantify immune cells in Figure 2f, 3c, 4d, 4e, Supplementary Figures 8a, 8b, and 11, and **(b)** T cell inhibitory receptors used to characterize T cells used in Figures 2g, Supplementary Figures 6a, 6b, and 8c are shown. Gating for PD-1, TIM-3, LAG-3, and CTLA-4 on activated CD8 T cells ($CD45^+THY1.2^+CD8^+CD44^+$) was determined by fluorescence minus one controls.
