# Supplementary material for: Syngeneic animal models of tobacco-associated oral cancer reveal the activity of in situ anti-CTLA-4
Source: Nat Commun. 2019 Dec 5;10:5546. doi: 10.1038/s41467-019-13471-0 (PMC6895221; doi:10.1038/s41467-019-13471-0)
Supplement: Supplementary file 2 — Description of Additional Supplementary Files [file 41467_2019_13471_MOESM2_ESM.docx]

**Description of Supplementary Files**

**File Name:** Supplementary Data 1

**Description:** 4NQO variant calling results for 4MOSC1. Excel file listing variant calling results from DNA sequencing of 4NQO lesions. Column descriptions refer to Genomic Location (Location), Allele, Mutational Consequence, Impact, Symbol, Ensembl Gene, Feature_Type, Transcript Feature, Biotype, cDNA position, CDS_position, Protein position, Amino Acid Mutation, Codon Change, Strand, Transcript Support Level (TSL), Annotation Alternatively Splice Transcripts (APPRIS), and Sorting Intolerant From Tolerant (SIFT). Filter applied to the 4MOSC1-4 samples are used to show only mutations that result in amino acid changes.

**File Name:** Supplementary Data 2

**Description:** 4NQO variant calling results for 4MOSC2. Description of Excel file is as described in legend for Supplementary Data 1.

**File Name:** Supplementary Data 3

**Description:** 4NQO variant calling results for 4MOSC3. Description of Excel file is as described in legend for Supplementary Data 1.

**File Name:** Supplementary Data 4

**Description:** 4NQO variant calling results for 4MOSC4. Description of Excel file is as described in legend for Supplementary Data 1.
