# Supplementary material for: Syngeneic animal models of tobacco-associated oral cancer reveal the activity of in situ anti-CTLA-4
Source: Nat Commun. 2019 Dec 5;10:5546. doi: 10.1038/s41467-019-13471-0 (PMC6895221; doi:10.1038/s41467-019-13471-0)
Supplement: Supplementary file 7 — Reporting Summary [file 41467_2019_13471_MOESM7_ESM.pdf]

natureresearch

Corresponding author(s): J. Silvio Gutkind NCOMMS-19-16599A

Last updated by author(s): October 27, 2019

Reporting Summary

Nature Research wishes to improve the reproducibility of the work that we publish. This form provides structure for consistency and transparency in reporting. For further information on Nature Research policies, see Authors & Referees and the Editorial Policy Checklist .

Statistics

For all statistical analyses, confirm that the following items are present in the figure legend, table legend, main text, or Methods section.

n/a	Confirmed	
☐	☒	The exact sample size (*n*) for each experimental group/condition, given as a discrete number and unit of measurement
☐	☒	A statement on whether measurements were taken from distinct samples or whether the same sample was measured repeatedly
☐	☒	The statistical test(s) used AND whether they are one- or two-sided<br>*Only common tests should be described solely by name; describe more complex techniques in the Methods section.*
☐	☒	A description of all covariates tested
☐	☒	A description of any assumptions or corrections, such as tests of normality and adjustment for multiple comparisons
☐	☒	A full description of the statistical parameters including central tendency (e.g. means) or other basic estimates (e.g. regression coefficient) AND variation (e.g. standard deviation) or associated estimates of uncertainty (e.g. confidence intervals)
☐	☒	For null hypothesis testing, the test statistic (e.g. *F*, *t*, *r*) with confidence intervals, effect sizes, degrees of freedom and *P* value noted<br>*Give P values as exact values whenever suitable.*
☒	☐	For Bayesian analysis, information on the choice of priors and Markov chain Monte Carlo settings
☒	☐	For hierarchical and complex designs, identification of the appropriate level for tests and full reporting of outcomes
☐	☒	Estimates of effect sizes (e.g. Cohen's *d*, Pearson's *r*), indicating how they were calculated

Our web collection on statistics for biologists contains articles on many of the points above.

Software and code

Policy information about availability of computer code

Data collection	TCGA; Mouse Genome Project
Data analysis	https://www.mathworks.com/matlabcentral/fileexchange/38724-sigprofiler; Strelka2, Varscan2, GATK4 Mutect2; nSolver (V2.0); Qupath (V0.2.0), GraphPad software (V7), FlowJo (V10).

For manuscripts utilizing custom algorithms or software that are central to the research but not yet described in published literature, software must be made available to editors/reviewers. We strongly encourage code deposition in a community repository (e.g. GitHub). See the Nature Research guidelines for submitting code & software for further information.

Data

Policy information about availability of data

All manuscripts must include a data availability statement. This statement should provide the following information, where applicable:

- Accession codes, unique identifiers, or web links for publicly available datasets
- A list of figures that have associated raw data
- A description of any restrictions on data availability

All data generated or analysed during this study are included in this published article (and its supplementary information files). Accession codes from data generated have been provided in the manuscript text

Field-specific reporting

Please select the one below that is the best fit for your research. If you are not sure, read the appropriate sections before making your selection.

Life sciences study design

All studies must disclose on these points even when the disclosure is negative.

Sample size	Sample sizes were chosen based on the historical data of the variability of tumor growth and treatment response observed. Sample size was determined to be adequate based on the consistency of measurable differences within and between groups.
Data exclusions	No data were excluded from these analyses.
Replication	Every experiment was replicated at least three with near-identical results.
Randomization	Based on the tumor volumes on the first day of treatment, tumor bearing mice were randomly assigned to treatment groups such that each treatment group or time point/treatment group had the same average tumor volume.
Blinding	The data presented did not require the use of blinding. Data reported for mouse experiments were not subjective but rather based on quantitative analyses.

Reporting for specific materials, systems and methods

We require information from authors about some types of materials, experimental systems and methods used in many studies. Here, indicate whether each material, system or method listed is relevant to your study. If you are not sure if a list item applies to your research, read the appropriate section before selecting a response.

Materials & experimental systems

n/a	Involved in the study	
☐	☒	Antibodies
☐	☒	Eukaryotic cell lines
☒	☐	Palaeontology
☐	☒	Animals and other organisms
☒	☐	Human research participants
☒	☐	Clinical data

Methods

n/a	Involved in the study	
☒	☐	ChIP-seq
☐	☒	Flow cytometry
☒	☐	MRI-based neuroimaging

Antibodies

Antibodies used	All flow cytometry antibodies from BioLegend, San Diego, CA: CD45 (30-F11), CD3 (145-2C11), CD8a (53-6.7), CD4 (RM4-4), NK1.1 (PK136). CD24 (M1/69), MHCII (M5/114.15.2), Ly6-G (1A8), Ly6-C (HK1.4), F4/80 (T45-2342), CD103 (2E7), CD11b (M1/70), CD11c (HL3), PD-1 (29F.1A12), TIM-3 (B8.2C12), CD44 (IM7), LAG-3 (C9B7W) and CTLA-4 (UC10-4B9).<br>All therapeutic antibodies from Bio X Cell (West Lebanon, NH, USA): PD-1 antibody (clone J43, catalog #BE0033-2), CTLA-4 antibody (clone 9H10, catalog #BP0131), isotype antibody (catalog # BE0091) and CD8 depletion antibody (Clone YTS 169.4, catalog #BE0117).<br>Immunofluorescence antibodies: CK5 (Fitzgerald, 20R-CP003), CD8 (abcam, ab22378), and FoxP3 (Cell Signaling Technology, #D608R).
Validation	All antibodies were validated by the supplier (BioLegend) and were checked in the lab by comparing to the manufacturer's or in-house results. Statement from BioLegend: BioLegend antibodies undergo an extensive series of testing to ensure quality at every step in the manufacturing process, as well as maintaining quality after the sale. Statement from Bio X Cell: Our InVivoPlus™ antibodies feature all the great qualities of our InVivoMab™ antibodies. The InVivoPlus™ versions of our products are structurally and functionally identical to the InVivoMab™ versions with the added benefit of additional QC measures. InVivoPlus™ antibodies are screened for murine pathogens using ultrasensitive qPCR, screened for protein aggregation via dynamic light scattering, feature advanced binding validation via flow cytometry, ELISA, and/or Western blot, and are guaranteed to contain less than 1 endotoxin unit per milligram. Our InVivoPlus™ line of antibodies are designed to exceed the strict demands and rigorous standards required for in vivo work at any research organization. For CK5 (Fitzgerald, 20R-CP003) , please see the information in this link, https://www.fitzgerald-fii.com/cytokeratin-5-antibody-20r-cp003.html. And from the literature, this antibody has been used in the following: PLoS ONE on 20 June 2019 by Beeler, J. S., Marshall, C. B., et al.p73 regulates epidermal wound healing and induced keratinocyte programming. And The Journal of Biological Chemistry on 17 July 2015 by Huang, Y., Hamana, T., et al. Prostate Sphere-forming Stem Cells Are Derived from the P63-expressing Basal Compartment. Statement from abcam: Antibody specificity is confirmed by looking at cells that either do or do not express the target protein within the same tissue. Initially, our scientists will review the available literature to determine the best cell lines and tissues to use for validation. We then check the protein expression by IHC/ICC to see if it has the expected cellular localization (Figure 3). If the localization of the signal is as expected, this antibody will pass and is considered suitable for use in IHC/ICC. We use a variety of methods, including staining multi-normal human tissue microarrays (TMAs), multi-tumor human TMAs, and rat or mouse TMAs during antibody development. These high-throughput arrays allow us to check many tissues at the same time, providing uniformly as all tissues are exposed to the exact same conditions. Statement from Cell Signaling Technology: https://www.cellsignal.com/contents/our-approach-antibody-validation-principles/antibody-validation-for-immunofluorescence/ourapproach-validation-if

Eukaryotic cell lines

Policy information about cell lines

Cell line source(s)	Cell lines were generated in-house.
Authentication	Authentication was done by genomic sequencing analysis.
Mycoplasma contamination	All cell lines are frequently tested for mycoplasma contamination. No presence of mycoplasma was found according to Mycoplasma Detection Kit-QuickTest from Biomake (Houston, TX, USA).
Commonly misidentified lines (See ICLAC register)	No commonly misidentified cells were used.

Animals and other organisms

Policy information about studies involving animals; ARRIVE guidelines recommended for reporting animal research

Laboratory animals	Female C57Bl/6 mice (4-6 weeks of age and weighing 16-18g) were purchased from Charles River Laboratories (Worcester, MA, USA). Foxp3DTR mice (Stock No. 016958) were purchased from The Jackson Laboratory and bred in-house. All the animal studies using HNSCC tumor xenografts and oral carcinogenesis studies were approved by the Institutional Animal Care and Use Committee (IACUC) of University of California, San Diego, with protocol ASP #S15195. Housing condition information is written in the manuscript.
Wild animals	Study did not involve wild animals.
Field-collected samples	Study did not involve samples from the field.
Ethics oversight	All studies were approved by the Institutional Animal Care and Use Committee (IACUC) of University of California, San Diego, and mouse procedures were performed following ACP guidelines.

Note that full information on the approval of the study protocol must also be provided in the manuscript.

Flow Cytometry

Plots

Confirm that:

- [x] The axis labels state the marker and fluorochrome used (e.g. CD4-FITC).
- [x] The axis scales are clearly visible. Include numbers along axes only for bottom left plot of group (a 'group' is an analysis of identical markers).
- [x] All plots are contour plots with outliers or pseudocolor plots.
- [x] A numerical value for number of cells or percentage (with statistics) is provided.

Methodology

Sample preparation	Tumors were dissected, minced, and re-suspended in complete media (DMEM with 10% FBS and 1% antibiotics) supplemented with Collagenase-D (lmg/mL; Roche) and incubated at 3TC for 30 minutes with shaking to form a single-cell suspension. Tissue suspensions were washed with fresh media and passed through a 100-µm strainer.
Instrument	BD LSRFortessa
Software	FlowJo
Cell population abundance	Cells were not sorted in this study.
Gating strategy	Single cells were gated from FSC/SSC (height/width) and live/dead cells were discriminated using BD HorizonTM Fixable Viability Stain 510. Cells were further characterized according to the following characteristics: cytotoxic T cells (CD45+ Thyl.2+CD8+), helper T cells (CD45+ Thyl.2+CD4+), Treg (CD45+ Thy1.2+CD4+FOXP3+), NK cells (CD45+ Thyl.2-NKl.1 +), macrophages (CD45 + Thyl.2-NK1.1-CDllb+CDllc-LY6ClowLY6GlowCD24+F4/80+), PMN-MDSCs (CD45+ Thyl.2-NK1.1-CDllb+CDllc-LY6ClowLY6G+), and M-MDSCs (CD45+ THyl.2-NK1.1-CDllb+CDllc-LY6C+LY6Glow).

- [x] Tick this box to confirm that a figure exemplifying the gating strategy is provided in the Supplementary Information.
